# Ubiquitination-mediated molecular pathway alterations in human lung squamous cell carcinomas identified by quantitative ubiquitinomics

**DOI:** 10.3389/fendo.2022.970843

**Published:** 2022-09-15

**Authors:** Xianquan Zhan, Miaolong Lu, Lamei Yang, Jingru Yang, Xiaohan Zhan, Shu Zheng, Yuna Guo, Biao Li, Siqi Wen, Jiajia Li, Na Li

**Affiliations:** ^1^ Shandong Key Laboratory of Radiation Oncology, Shandong Cancer Hospital and Institute, Shandong First Medical University and Shandong Academy of Medical Sciences, Jinan, China; ^2^ Medical Science and Technology Innovation Center, Shandong First Medical University, Jinan, China; ^3^ Key Laboratory of Cancer Proteomics of Chinese Ministry of Health, Xiangya Hospital, Central South University, Changsha, China

**Keywords:** lung squamous cell carcinoma, ubiquitination, ubiquitinated protein, signaling pathway, ubiquitin-proteasome system, biomarker

## Abstract

Abnormal ubiquitination is extensively associated with cancers. To investigate human lung cancer ubiquitination and its potential functions, quantitative ubiquitinomics was carried out between human lung squamous cell carcinoma (LSCC) and control tissues, which characterized a total of 627 ubiquitin-modified proteins (UPs) and 1209 ubiquitinated lysine sites. Those UPs were mainly involved in cell adhesion, signal transduction, and regulations of ribosome complex and proteasome complex. Thirty three UPs whose genes were also found in TCGA database were significantly related to overall survival of LSCC. Six significant networks and 234 hub molecules were obtained from the protein-protein interaction (PPI) analysis of those 627 UPs. KEGG pathway analysis of those UPs revealed 47 statistically significant pathways, and most of which were tumor-associated pathways such as mTOR, HIF-1, PI3K-Akt, and Ras signaling pathways, and intracellular protein turnover-related pathways such as ribosome complex, ubiquitin-mediated proteolysis, ER protein processing, and proteasome complex pathways. Further, the relationship analysis of ubiquitination and differentially expressed proteins shows that ubiquitination regulates two aspects of protein turnover - synthesis and degradation. This study provided the first profile of UPs and molecular networks in LSCC tissue, which is the important resource to insight into new mechanisms, and to identify new biomarkers and therapeutic targets/drugs to treat LSCC.

## Introduction

Lung squamous cell carcinoma (LSCC) is closely related to smoking (1), which is 25%-30% of non-small cell lung cancer (NSCLC), and >70% LSCC patients were diagnosed in late stage (2, 3), with high mortality (~0.4 million/year) in the world (4). Although the incidence has decreased due to tobacco control and changes in lifestyle in recent decades, LSCC still brings huge burdens to society (5). In terms of treatment, radical thoracic surgery is preferred for early-stage cases of LSCC; however, unfortunately the proportion of this type of patients is very small. Besides, chemotherapy and radiotherapy are also indispensable treatments, especially in advanced (or metastatic) LSCC subpopulation (6). Recent years, although some encouraging targeted drugs such as anaplastic lymphoma kinase inhibitors and epithelial growth factor receptor (EGFR) tyrosine kinase inhibitors have been used to significantly improve the therapy of late-stage lung adenocarcinoma (4), there is still lack of FDA-approved targeted therapy for advanced LSCC patients. For most patients with advanced LSCC, first-line standard chemotherapy is a 4-6-cycle platinum-based third-generation cytotoxic drug with an effective rate of 20%-30%. The median survival time (MST) is 8-10 months, the time to progression (TTP) is 3-5 months (7). In addition, LSCC still lacks universally accepted biomarkers for early diagnosis and prognostic evaluation. Obviously, the current predictive, preventive, and personalized medicine (PPPM) of LSCC is still unmet; therefore, it is necessary to clarify molecular mechanism and discover new biomarkers and therapeutic targets/drugs to manage LSCC.

Protein ubiquitination is an important molecular event, which covalently link the C-terminus of ubiquitin (8.5 kDa, 76 amino acids, coded by four genes RPS27A, UBA52, UBC, and UBB) to the ϵ-amino group at lysine residue in a protein (8, 9). Ubiquitination is a multi-step reaction process, which is catalyzed by enzymes E1, E2, and E3 (8). Protein ubiquitinations are classified into monoubiquitination that means one ubiquitin in one protein, multiubiquitination that means several ubiquitination sites in a protein and each ubiquitination site links one ubiquitin, and polyubiquitination that means a polyubiquitin chain to be added to lysine residues or N-terminus of the previous ubiquitin (10); and different types of ubiquitinations have different biological functions (11). Also, there are seven lysine residues in an ubiquitin, which make polyubiquitin chain more complicated. Moreover, ubiquitination process can be reversed, which is catalyzed by more than 100 deubiquitination enzymes (12). The reversible ubiquitination reaction regulates multiple biological processes including protein degradation (13), and associated with a wide range of diseases including cancers, and inflammatory diseases (14). Ubiquitin-proteasome system (UPS) is the important protein degradation pathway, which has important roles in tumorigenesis. Targeting the UPS system is the promising anti-tumor strategies. Thereby, several effective anti-tumor drugs targeting UPS have been approved by FDA, including bortezomib (15), carfilzomib (16), thalidomide, pomalidomide, and lenalidomide (17), for different cancer treatment. At present, LSCC still lacks specific early diagnostic biomarkers, and in addition to surgery, radiotherapy, and chemotherapy, there is lack of effective molecular therapeutic drugs. Therefore, the study of ubiquitinome might have important scientific merits for discovery of effective biomarkers and novel therapeutic targets/drugs for LSCC.

Liquid chromatography in combination with tandem mass spectrometry (LC-MS/MS) is an effective method to identify ubiquitination sites and quantify the abundance of ubiquitination (18). However, endogenous ubiquitination is low abundant event, it is necessary to enrich the ubiquitinated tryptic peptides prior to MS/MS analysis. The commercial anti-K-ϵ-GG antibodies is able to effectively enrich ubiquitinated tryptic peptides (19). For antibody-based enrichment of ubiquitinated tryptic peptides, it is the best to avoid any salt, acid, and basic factors to negatively impact this enrichment. Compared to the isotope-labeling quantitative proteomics such as iTRAQ (isobaric tags for relative and absolute quantification) and TMT (tandem mass tags) that introduce above interfered factors to negatively impact antibody-antigen reaction, label-free quantitative proteomics is a preferred approach to identify and quantify endogenous ubiquitination, which does not label the analyzed protein component in a proteome (20) but quantify the protein with area under the curve (AUC) and signal intensity in the MS spectrum, and spectral counting based on MS/MS analysis (21). Thus, anti-ubiquitin antibody in combination with label-free LC-MS/MS is an effective quantitative ubiquitinomics, which has identified up to 10,000 ubiquitination sites in an experiment (22). For lung cancer analysis, currently researchers mainly focused on the study on ubiquitinome of lung cancer cells (23, 24), and these studies were not based strictly on LSCC cell lines.

This study used anti-ubiquitin antibody coupled with label-free LC-MS/MS to detect, identify, and quantify ubiquitinated proteins (UPs) and ubiquitination sites in fresh human LSCC tissues compare to controls. Gene ontology (GO) enrichment analysis was used to determine the functional characteristics of UPs, and Kyoto Encyclopedia of Genes and Genomes (KEGG) enrichment analysis was used to mine the ubiquitination-involved pathway networks. This study provides the holistic profile of ubiquitination in LSCC tissue, and underscores ubiquitination may promote the occurrence and development of LSCC by affecting the intracellular protein turnover, and paving the way for further research on specific ubiquitination regulatory mechanisms.

## Materials and methods

### Tissue samples and protein preparation

Human LSCC tissues (n =5) and tumor-adjacent control (TAC) tissues (n=5) (**Supplementary Table 1**; these samples were at early stage of LSCC) were removed by thoracic surgery, and stored in –80°C, for protein extraction. A total of 750 mg LSCC tissues (n = 5; 150 mg per patient) and 750 mg TAC tissues (n=5; 150 mg per patient) derived from 5 patients were homogenized, after removal of blood contamination, in solution that included 2 M thiourea, 7 M urea, 1 mM phenylmethyl-sulfonyl fluoride (PMSF), and 100 mM dithiothreitol (DTT); followed by sonication (80 W, 10 s, and interval 15 s; 10 x), and centrifugation at 4°C (15,000xg, and 20 min). The supernatant was the extracted proteins, whose protein content was determined with Brandford assay.

### Preparation of tryptic peptides and enrichment of ubiquitinated tryptic peptides

The protein sample (LSCC; TAC) was reduced with final concentration of 10 mM DTT (37 °C, 1.5 h), and alkylated with final concentration of 50 mM iodoacetamide (30 min, dark). The mixture was diluted to 2 M uranyl acetate with 50 mM Tris-HCl (pH 8.0), follwed by addition of trypsin (1:50 for the ratio of trypsin to protein), and incubation (15-18 h; 37 °C) to digest the proteins. Then, the digestion reaction was stopped with addition of trifluoroacetic acid (TFA) to adjust pH ≤ 3. The tryptic peptide mixture was desalted, and lyophilized. The lyophilized tryptic peptides were redisolved in 1.4 mL solution that contained 50 mM NaCl, 10 mM Na_2_HPO_4_, and 50 mM MOPS/NaOH pH 7.2, followed by addition of anti-K-ϵ-GG antibody beads [PTMScan ubiquitin remnant motif (K-ϵ-GG) kit, Cell Signal Technology], incubation (1.5 h, 4 °C), and centrifugation (30 s, 2,000×g). The beads with antibody-binding ubiquitinated peptides were washed for 3 times with 1 mL solution that contained 50 mM NaCl, 10 mM Na_2_HPO_4_, and 50 mM MOPS/NaOH pH 7.2, followed by washing with water for 3 times. The ubiquitinated tryptic peptides were eluted with 40 μL 0.15% TFA, followed by centrifugation (30 s, 2,000×g), and desaltation with C18 STAGE Tips.

### LC-MS/MS analysis of enriched ubiquitinated peptides

The prepared ubiquitinated tryptic peptides were treated with a reverse-phase trap column (nanoViper C18, 2 cm x 100 μm), and then were separated in a reverse-phase analytical column (C18, 10 cm length x 75 μm i.d., and 3 μm resin) with buffers A (0.1% formic acid) and B (0.1% formic acid + 84% acetonitrile) for 2 h (flow-rate =300 nl/min) on the Q Exactive mass spectrometer (Thermo Scientific) to obtain MS/MS raw data. MaxQuant software (version 1.5.3.17) was used to search MS/MS data against human protein database uniprot_human_156639_20170105.fasta, with database search parameters (4 missed cleavages, trypsin, 6 ppm for MS, 20 ppm for MS/MS, GlyGly at Lys, acetylation at protein N-term, oxidation at Met, and carbamidomethyl at Cys). The peptide was determined by MS/MS data with false discovery rate (FDR) ≤0.01, ubiquitination site with FDR ≤0.01, and protein with FDR ≤0.01. The abundance of ubiquitination was determined with MaxQuant label-free calculation based on peptide intensity and peptide counting.

### Bioinformatics and statistical analysis

The functional characteristics of the identified UPs, including biological processes (BPs), molecular functions (MFs), and cellular components (CCs) were analyzed with DAVID software (version 6.8, https://david.ncifcrf.gov/) (25). Ubiquitination-mediated signaling pathways were analyzed with the KEGG tool KOBAS (http://kobas.cbi.pku.cn) (26). The protein-protein interaction (PPI) networks that UPs are involved in were analyzed with STRING software (https://string-db.org/; version 10.0) (27), with statistical significance of a combined score > 0.4, in the Cytoscape program (version 3.6.0) (28). Also, molecular complex detection (MCODE) in the Cytoscape program was used to identify significant modules in the PPI networks with cutoff values (max depth=100, node score=0.2, degree=2, k-score=2, and MCODE score>5) (29). The hub nodes of PPI network were determined with cytoHubba (version 0.1) in Cytoscape package through 6 different topological algorithms (30), including Maximal Clique Centrality (MCC; cutoff score=100 with this node that had at least 5 molecules linked), Maximum Neighborhood Component (MNC; cutoff score=10 with this node that had at least 5 molecules linked), Degree (cutoff score=10 with this node that had at least 5 molecules linked), Edge Percolated Component (EPC; cutoff score=10 with this node that had at least 5 molecules linked), Closeness (cutoff=150 with this node that had at least 5 molecules linked), and Raliality (cutoff score=5 with this node that had at least 5 molecules linked). The final hub nodes were identified with overlapped analysis of six sets of hub nodes that were derived from 6 topological algorithms. The transcriptomic data (level 2 count data) and corresponding clinical survival data of LSCC patients were obtained from TCGA database with R-languages “RTCGA.mRNA” (version 1.12.0) and “RTCGA.clinical” (version 20151101.14.0), respectively. The survival analysis was performed with R-languages “survival” (version 2.44-1.1) and “survminer” (version 0.4.6).

**Figure 1:**
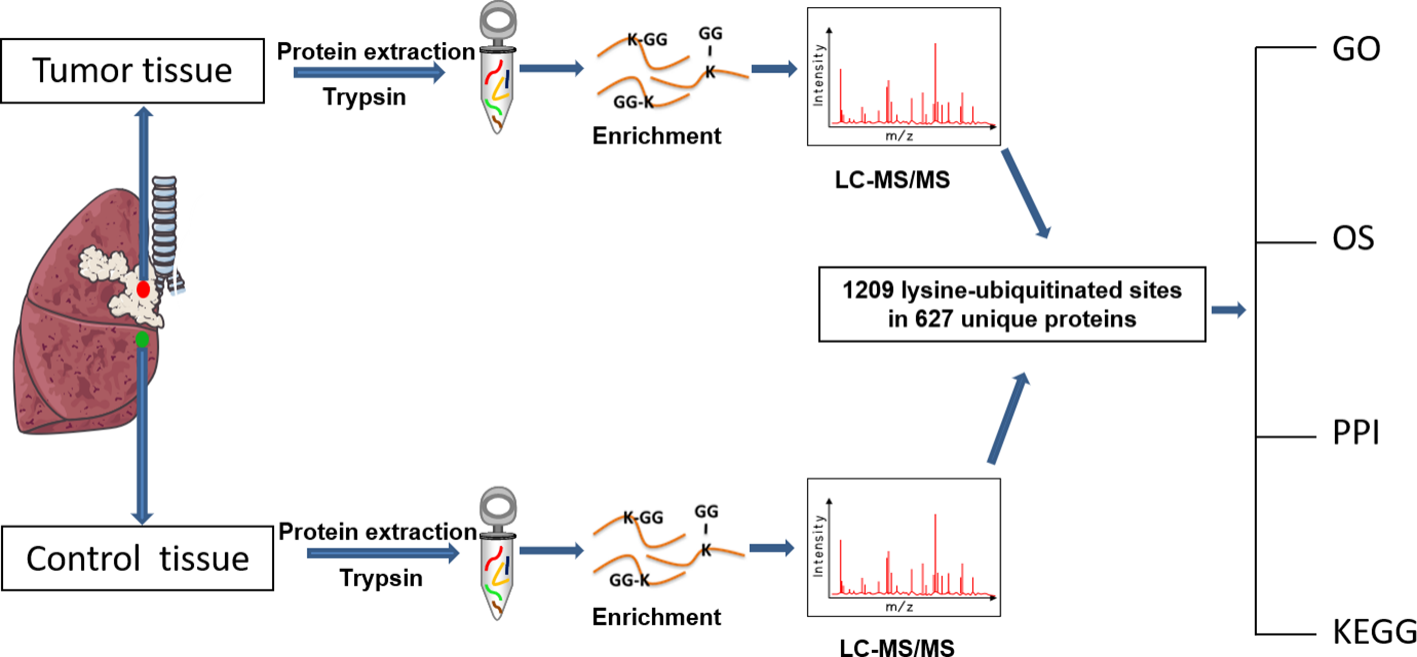
Experimental flow-chart and bioinformatics analysis that were used in this study.

The overall flow-chart to identify and analyze the ubiquitination profile and the corresponding functional characteristics in LSCC was summarized (**Figure 1**).

## Results

### Ubiquitination profile in human LSCC

**Figure 2:**
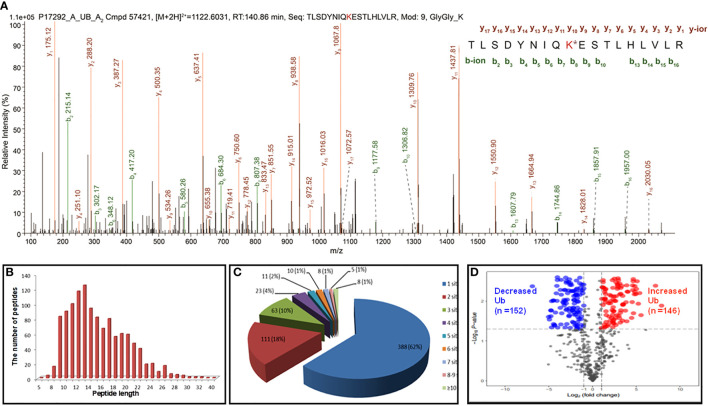
Representative MS/MS spectra and holistic characteristics of ubiquitination sites in LSCC. **(A)** The identified ubiquitinated peptide ^55^TLSDYNIQK^*^ESTLHLVLR^72^ from 40S ribosomal protein S27a (RPS27A, P62987). K* = ubiquitinated lysine residue. **(B)** The length distribution of identified ubiquitinated peptides. **(C)** Pie-chart demonstrating the number of ubiquitination sites per protein. **(D)** Volcano plot demonstrated the changes in the degree of ubiquitination at a special lysine site among 642 ubiquitination sites (T+/N+) according to the fold change and p value between the LSCC and control groups. X axis: the log of the fold change between the two conditions (log base 2). Y axis: the negative log of the p value (log base 10). Red dot indicates that the degree of ubiquitination of the site is significantly increased (Ratio T/N>2.0; p<0.05) and blue indicates significantly decreased (Ratio T/N < 0.5; p<0.05).

In total, 627 UPs and 1209 ubiquitination sites were identified with MS/MS data, which referred to 1086 ubiquitinated tryptic peptides, in human LSCC tissues (**Supplementary Table 2**). For example, the MS/MS spectrum of tryptic peptide ^55^TLSDYNIQK^*^ESTLHLVLR^72^ ([M+2H]^2+^ *m/z* =1122.6031, RT = 140.86 min) was derived from 40S ribosomal protein S27a (RPS27A; P62987) (**Figure 2A**), with high S/N (signal to noise) ratio, excellent y-ion (y_1_-y_17_) and b-ion (b_2_-b_10_, b_13_-b_16_) series, localization of ubiquitination site at residue K_63_, and a decreased ubiquitination level at residue K63 (Ratio T/N = 0.425; P=0.0529) in LSCCs compared to TAC controls (**Supplementary Table 2**). In addition, another six ubiquitination sites were also identified in this RPS27A (P62987) in LSCC tissues compare to TAC tissues, including ubiquitinations at residues K6 (Ratio T/N = 0.271; P=5.07E-03), K11 (Ratio T/N=0.496; P=1.28E-02), K27 (Ratio T/N=3.812; P=4.53E-05), K29 (Ratio T/N=0.933; P=8.55E-01), K33 (Ratio T/N=2.903; P=3.75E-04), and K48 (Ratio T/N=0.839; P=5.47E-01) (**Supplementary Table 2**). It clearly demonstrated that 7 ubiquitination sites were identified in this RPS27A, with decreased ubiquitination levels at residues K6, K11, K29, K48, and K63 (of them, significantly decreased level at residues K6 and K11), and significantly increased ubiquitination levels at residues K27 and K33. This is an interesting result, because, in the same protein, the ubiquitination level at some Lys sites were decreased (K6, K11, K29, K48, and K63), and whereas the ubiquitination levels at some Lys sites were increased (K27 and K33). This phenomenon might be easily explained from the angle of proteoforms encoded by the same gene RPS27A. Another reason might be due to the kinetics change of ubiquitin linkages in LSCC because there are four ubiquitin-encoding genes, including UBB, UBC, UBA52 (which encodes RPL40), and UBA80 (also known as RPS27A; RPS27A encodes a fusion protein consisting of ubiquitin at the N terminus and ribosomal protein S27a at the C terminus), and all of which produce the identical ubiquitin protein; if S27a protein does not change, but the ubiquitin chain linking pattern increases or decreases, it is not due to the RPS27A gene alone; thereby thus results in the alteration of ubiquitin ligation dynamics mediated by ubiquitin ligases in LSCC.

For 1086 ubiquitinated tryptic peptides, their lengths were in the range of 5-40 amino acids (**Figure 2B**). Analysis of the number of ubiquitination sites in human LSCC UPs found that the number of putative ubiquitination sites per protein was arranged from 1 to 15. A total of 388 (388/627 = 62%) UPs had only one ubiquitination site, 18% (111/627) UPs had two sites, 10% (63/627) UPs had three sites, 4% (23/627) UPs had four sites, and 7% (42/627) UPs had five sites (**Figure 2C**). A total of 1133 (1133/1209 = 93.7%) ubiquitination sites had quantitative information, including 642 (642/1133 = 56.7%) sites with T+/N+, 395 (395/1133 = 34.9%) with T+/N-, and 96 (96/1133 = 8.4%) with T-/N+; and among these 1133 sites, 699 (61.7%) sites had increased ubiquitination levels and 434 (38.3%) sites had decreased ubiquitination levels in LSCC compared to TAC tissues. Another 76 (76/1209 = 6.3%) sites did not have quantitative information between LSCC and TAC tissues. Here showed the distribution of intensity changes of these 642 ubiquitination sites (T+/N+), including 146 sites had significantly increased ubiquitination levels (Ratio T/N >2; p<0.05), and 152 sites had significantly decreased ubiquitination levels (Ratio T/N <0.5; p<0.05) (**Figure 2D**), the corresponding detailed information was listed in **Supplementary Table 2**.

### Functional characteristics of indentified UPs in LSCC

**Figure 3:**
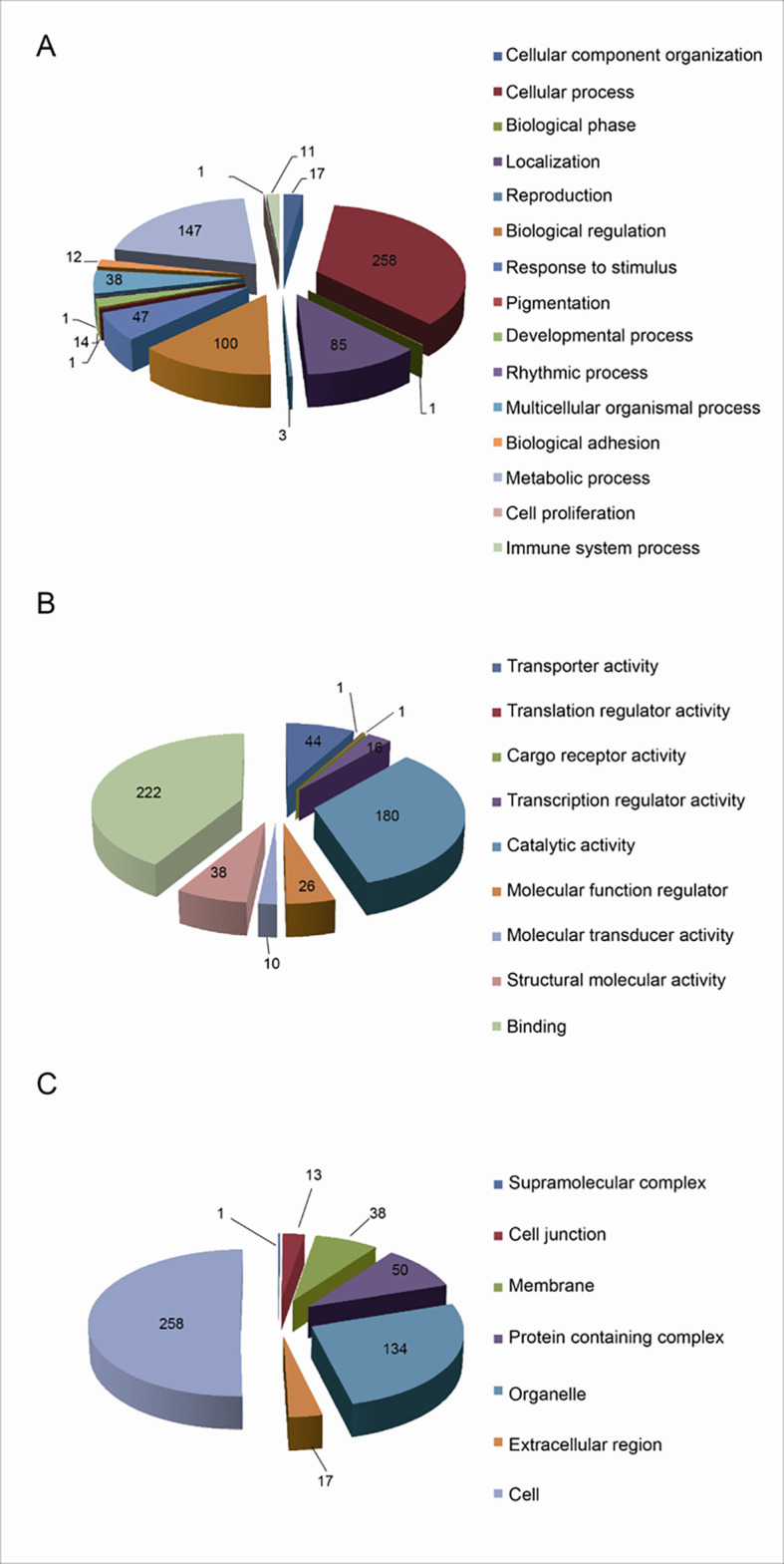
Functional characteristics of 627 Ups identified with GO enrichment analysis. **(A)** The BP profile of UPs. **(B)** The MF profile of UPs. **(C)** The CC profile of UPs.

The functional characteristics of 627 UPs were enriched with GO analysis, including BPs, MFs, and CCs. For BP analysis, UPs were mainly enriched in cellular process (258 UPs), metabolic process (147 UPs), biological regulation (100 UPs), and localization (85 UPs) (**Figure 3A**). For MF analysis, UPs were mainly enriched in binding activity (222 UPs), catalytic activity (180 UPs), transporter activity (44 UPs), structural molecule activity (38 UPs), molecular function regulator (26 UPs), and transcription regulator activity (16 UPs) (**Figure 3B**). For CC analysis, UPs were mainly enriched in cell (258 UPs), organelle (134 UPs), protein-containing complex (50 UPs), membrane (38 UPs), extracellular region (17 UPs), and cell junction (13 UPs) (**Figure 3C**).

These BPs, MFs, and CCs were further grouped into 7 functional clusters (**Supplementary Table 3**), including cell adhesion, multiple signaling pathways that were closely related to tumorigenesis, such as Wnt signaling, tumor necrosis factor-mediated signaling, NF-kappa B signaling, assembly and functional regulation of proteasome, assembly and functional regulation of ribosome, haptoglobin hemoglobin complex, DNA damage recognition, and hemoglobin complex.

### Survival-related UPs in LSCC

**Figure 4:**
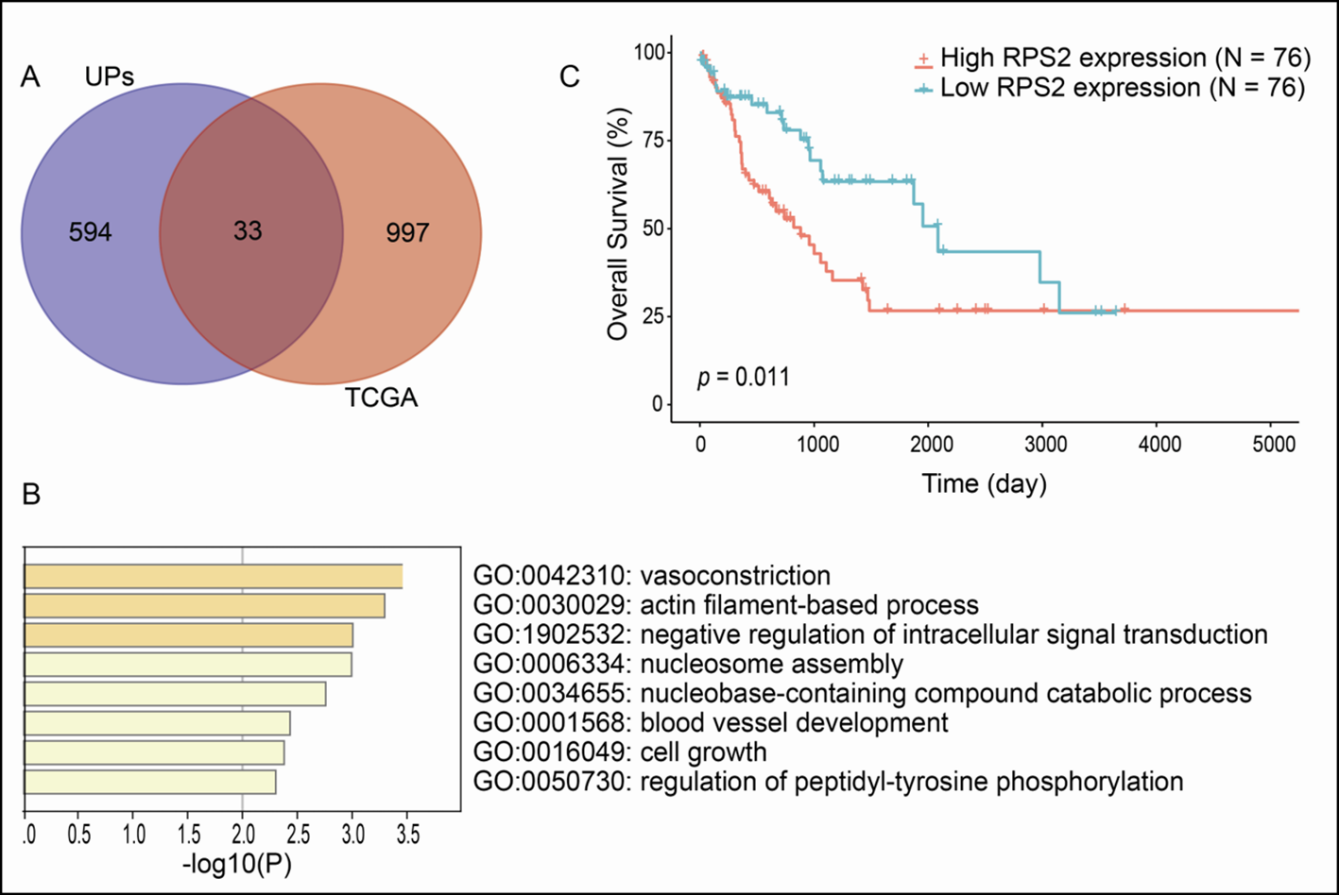
Overall survival-related UPs identified with overlapping analysis between 627 UPs and survival-related genes derived from TCGA database. **(A)** Venn diagram showed 33 molecules were associated with prognosis. **(B)** The biological processes involved in these prognostic related molecules. **(C)** Overall survival curve of RPS2.

A total of 1030 overall survival-related genes were obtained from LSCC data in TCGA database (**Supplementary Tables 4**-**6**). A total of 33 overlapped molecules (UPs; survival-related genes) were obtained when overlapping analysis was performed between the identified 627 UPs and 1030 overall survival-related genes (**Figure 4A**), which showed that those 33 overlapped molecules were survival-related UPs. GO enrichment analysis of these 33 survival-related UPs showed that these UPs were significantly involved in different BPs, and were closely related to tumorigenesis, including cell growth, blood vessel development, and regulation of intracellular signal transduction (**Figure 4B**). Among them, the higher expressions of 11 overlapped molecules (UPs; survival-related genes), including SPG20, PFN2, SET, CBX1, HISTIH1B, DSC3, ENO1, NONO, HISTIH4H, ACTA2 and PGK showed a better prognosis, which suggested their protective effects; while the higher expressions of 22 overlapped molecules (UPs; survival-related genes), including PRKCKBP, DDX5, ATP11A, EPB2, RPS2, ITGB1, TANK, GPRC5A, TRIM47, MVP, RNF213, SAP18, HM13, ICAM1, SLC16A4, LRSAM1, TM9SF3, CALD1, SYNPO, CLTB, CA1 and GJA5 demonstrated a worse prognosis, which suggested their tumor-promoting effects. Here showed the overall survival curve of RPS2 (**Figure 4C**), and RPS2 was further identified as a hub molecule.

### Ubiquitination-mediated molecular networks and network-based hub molecules derived from 627 UPs in LSCCs

**Figure 5:**
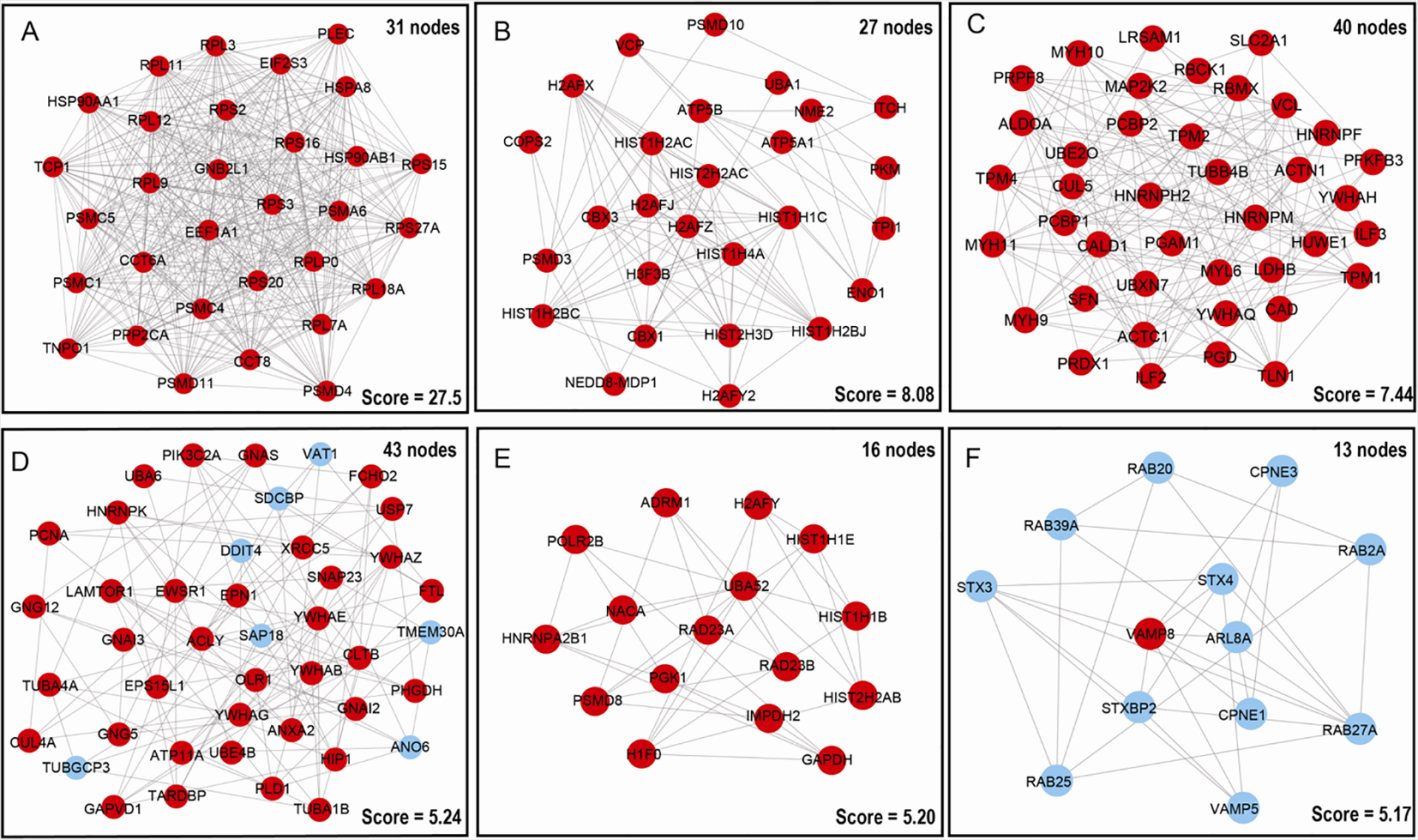
Molecular interaction modules and hub nodes based on PPI molecular networks of 627 UPs. **(A–F)** Six molecular interaction modules that meet screening criteria derived from the entire UPs PPI network. Red nodes represented the hub molecules. Blue node means it was not recognized as hub molecule due to low score.

To deeper understand cellular processes regulated by ubiquitination in LSCC, 627 UPs were input into STRING online software, 457/627 UPs were legible for PPI network analysis, which identified 6 significantly molecular networks (**Figures 5A–F** and **Supplementary Table 7**). The functional analyses of genes within each network were carried out with DAVID, which found that network 1 (31 nodes and MCODE score=27.55) was mainly involved in multiple proteasome and ribosomal subunits to regulate UPS and ribosome large subunits assembly, translation, protein processing, and intracellular signaling pathway; network 2 (27 nodes and MCODE score=8.08) was mainly involved in intranuclear regulation of gene expression; network 3 (40 nodes and MCODE score=7.44) was mainly referred to cell movement; network 4 (43 nodes and MCODE score=5.24) mainly participated in cell adhesion and membrane organization; network 5 (16 nodes and MCODE score=5.20) did not have significant biological processes; and network 6 (13 nodes and MCODE score=5.17) played important roles in protein transport and small GTPase-mediated signal transduction (**Supplementary Table 8**).

Moreover, 627 UPs were input into cytoHubba to determine hub molecules with 6 different topological algorithms, respectively; which identified 263 hub molecules with MCC, 232 with MNC, 268 with Degree, 278 with EPC, 347 with Closeness, and 334 with Raliality (**Supplementary Table 9**). Overlapped analysis of these six sets of hub molecules finally obtained 234 hub molecules (**Supplementary Table 9**). These finally determined 234 hub molecules were clustered into 5 functional categories, including cell-cell adhesion, ribosome-related biological process, proteasome-related biological process, cellular signaling pathways, and cellular energy metabolism (**Supplementary Table 10**). Interestingly, these results were also consistent with those results of GO enrichment analysis of 627 UPs (**Supplementary Table 3**).

### Ubiquitination-mediated signaling pathways in LSCC

**Figure 6:**
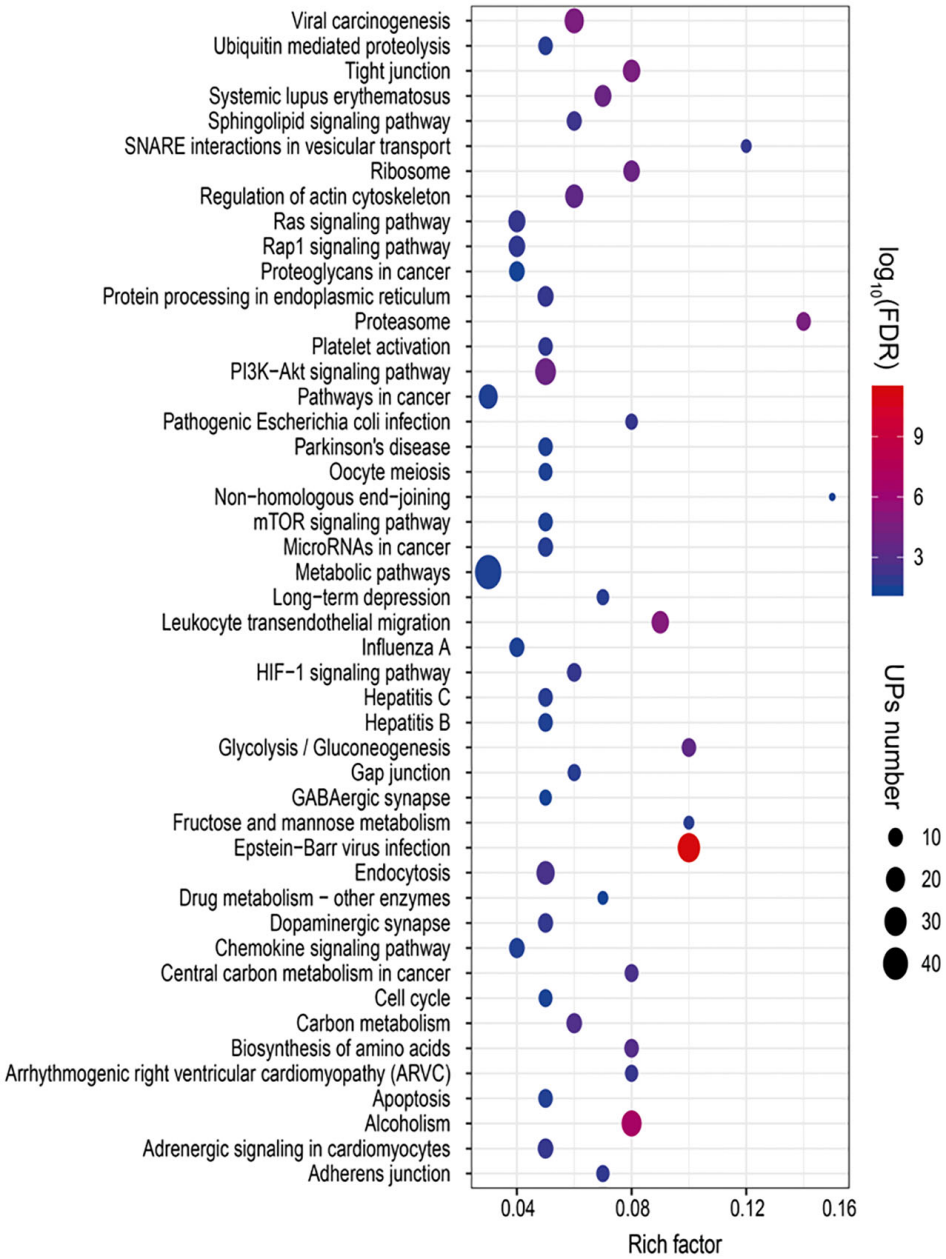
Statistically significant signaling pathways identified with KEGG pathway analysis of 627 UPs. A total of 47 KEGG pathways was significantly enriched from those UPs (p < 0.05; FDR < 0.05).

To reveal ubiquitination-mediated signaling pathway changes in LSCCs, 47 statistically significant pathways were revealed with KEGG pathway analysis of 627 UPs (**Figure 6** and **Supplementary Table 11**). Multiple signaling pathways were cancer-related pathways, including (i) ubiquitin-mediated proteolysis (10 UPs, and FDR =0.0184), ribosome complex (14 UPs, and FDR<0.01), ER-mediated protein processing (13 UPs, and FDR<0.01), proteasome complex (12 UPs, and FDR<0.01), and biosynthesis of amino acids (10UPs, and FDR<0.01), which was intracellular protein turnover-related pathways, and might disturb the synthesis-degradation balance of protein in tumorigenesis; (ii) PI3K-Akt signaling pathway (25 UPs, and FDR < 0.01), Ras signaling pathway (15 UPs, and FDR < 0.01), mTOR signaling pathway (10 UPs, and FDR = 0.03), HIF-1 signaling pathway (10 UPs, and FDR < 0.01), cell cycle (9 UPs, and FDR = 0.03), and apoptosis pathway (10 UPs, and FDR = 0.02), which might function in tumorigenesis; (iii) glycolysis/gluconeogenesis (10 UPs, and FDR<0.01), carbon metabolism (12 UPs, and FDR<0.01), central carbon metabolism in cancer (9 UPs, and FDR<0.01), Fructose and mannose metabolism (5 UPs, and FDR=0.019), metabolomic pathways (46 UPs, and FDR=0.031), drug metabolism-other enzymes (5 UPs, and FDR=0.0457), and sphingolipid signaling pathway (11 UPs, and FDR<0.01), which were obviously metabolism-related pathways; (iv) Tight junction (16 UPs, and FDR<0.01), adherens junction (8 UPs, and FDR=0.0126), gap junction (8 UPs, and FDR<0.01), and non-homologous end-joining (3 UPs, and FDR<0.0356), which were involved in cell adherens and junction; (v) endocytosis (18 UPs, and FDR<0.01), and SNARE interactions in vesicular transport (5 UPs, and FDR=0.0118). which might be substance transport-related pathways; (vi) dopaminergic synapse (11 UPs, and FDR=0.0115), and GABAergic synapse (7 UPs, and FDR=0.0415), which function in synapse pathway; and (vii) others pathways such as chemokine signaling pathway (12 UPs, and FDR=0.0287), Rap1 signaling pathway (14 UPs, and FDR=0.0124), proteoglycans in cancer (12 UPs, and FDR=0.0466), pathways in cancer (20 UPs, and FDR=0.0287), microRNAs in cancer (11 UPs, and FDR=0.0208), leukocyte transendothelial migration (16 UPs, and FDR<0.01), regulation of actin cytoskeleton (18 UPs, and FDR<0.01), and platelet activation (10 UPs, and FDR=0.0129). These ubiquitination-mediated cancer-related signaling pathways clearly demonstrated that ubiquitination played important roles in LSCC pathophysiological processes, and that ubiquitination was not only involved in the synthesis-degradation process of protein, but also in other multiple cancer-related signaling pathways.

**Figure 7:**
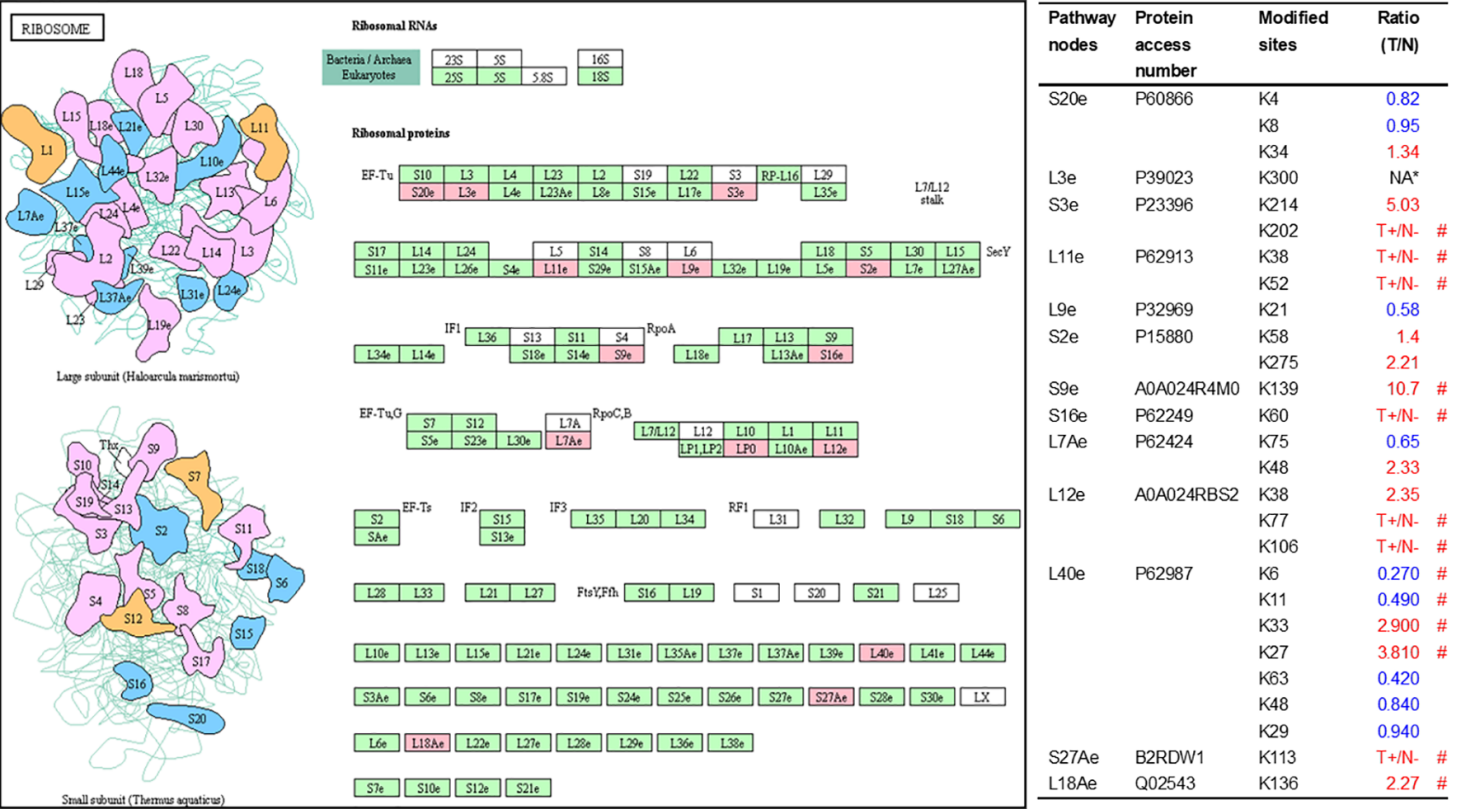
Ubiquitination-mediated ribosome pathway. Pink = the identified ubiquitinated subunits. Green=non-ubiquitinated subunits. K* = ubiquitinated lysine residue. Red indicates the degree of ubiquitination of this site is upregulated, and blue indicates downregulated. Ratio(T/N) = Ratio of tumor to control. T+/N- = ubiquitination only in tumor. NA* means no quantitative information in both tumor and control. # means statistically significantly altered ubiquitination level in tumor compared to controls.

**Figure 8:**
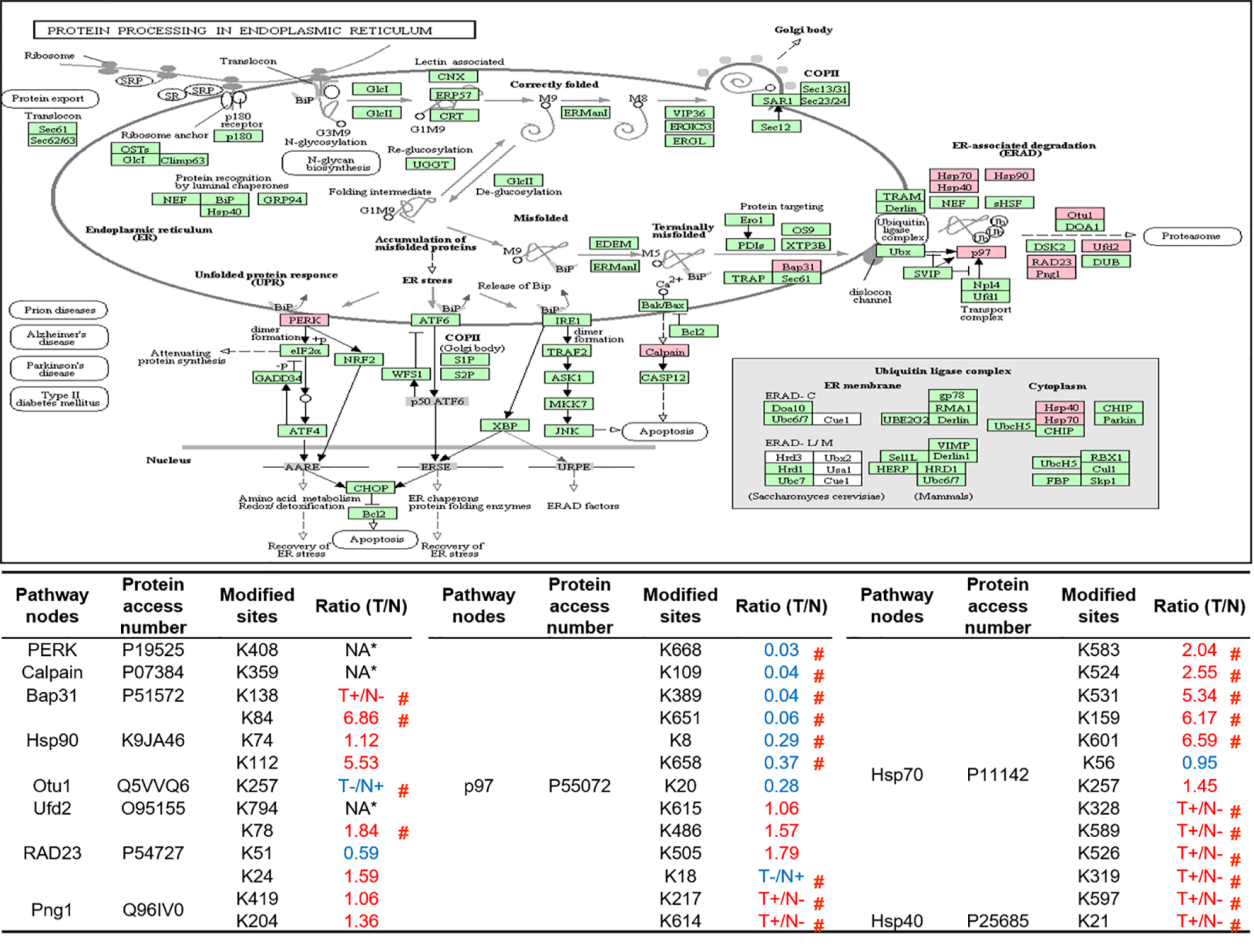
Ubiquitination-mediated endoplasmic reticulum protein processing pathway. Pink = the identified ubiquitinated subunits. Green=non-ubiquitinated subunits. K* = ubiquitinated lysine residue. Red indicates the degree of ubiquitination of this site is upregulated, and blue indicates downregulated. Ratio(T/N) = Ratio of tumor to control. T+/N- = ubiquitination only in tumor. T-/N+ = ubiquitination only in control. NA* means no quantitative information in both tumor and control. # means statistically significantly altered ubiquitination level in tumor compared to controls.

**Figure 9:**
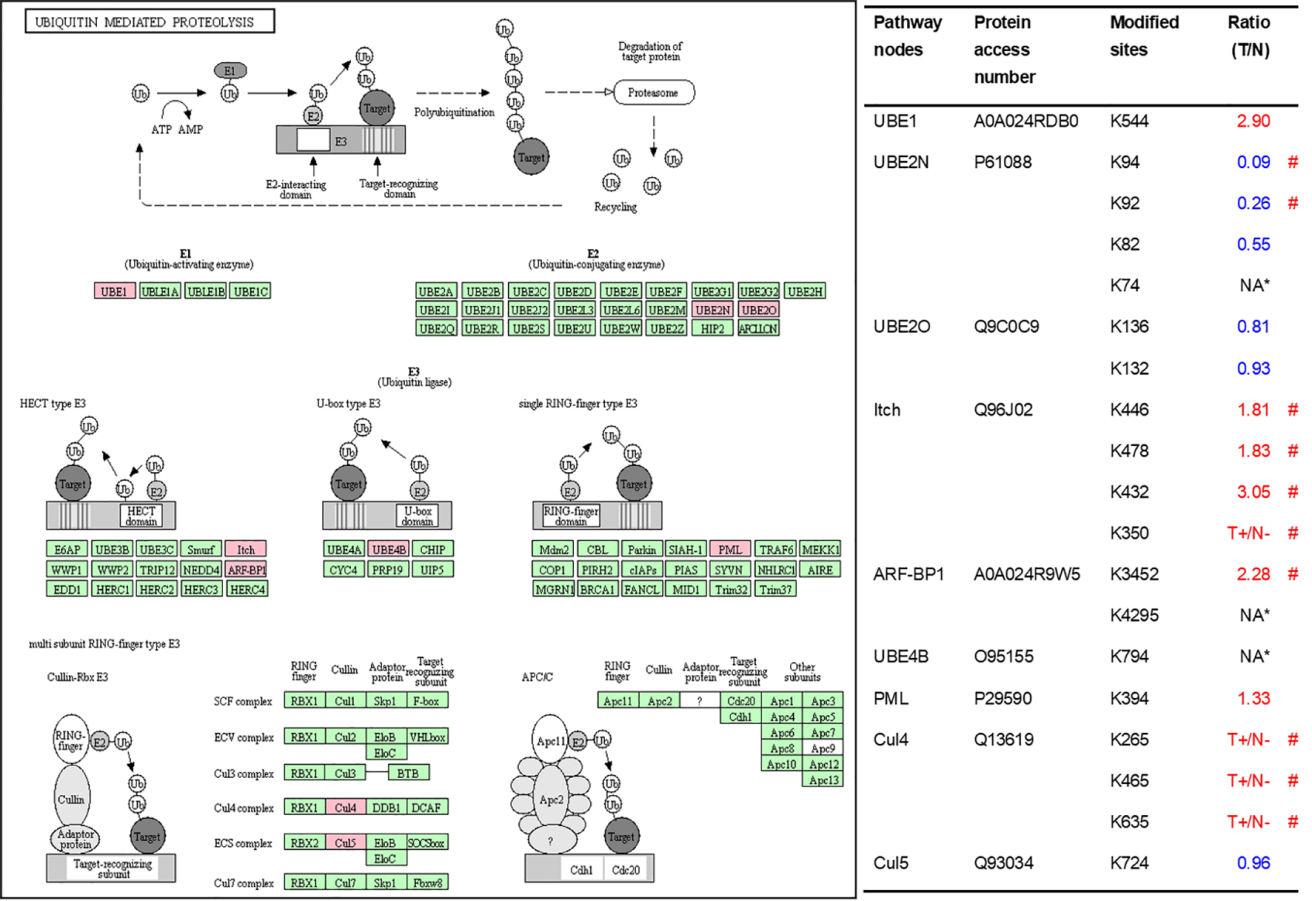
Ubiquitination-mediated UPS-related enzymes. Pink = the identified ubiquitinated subunits. Green = non-ubiquitinated subunits. K* = ubiquitinated lysine residue. Red indicates the degree of ubiquitination of this site is upregulated, and blue indicates downregulated. Ratio(T/N) = Ratio of tumor to control. T+/N- = ubiquitination only in tumor. NA* means no quantitative information in both tumor and control. # means statistically significantly altered ubiquitination level in tumor compared to controls.

**Figure 10:**
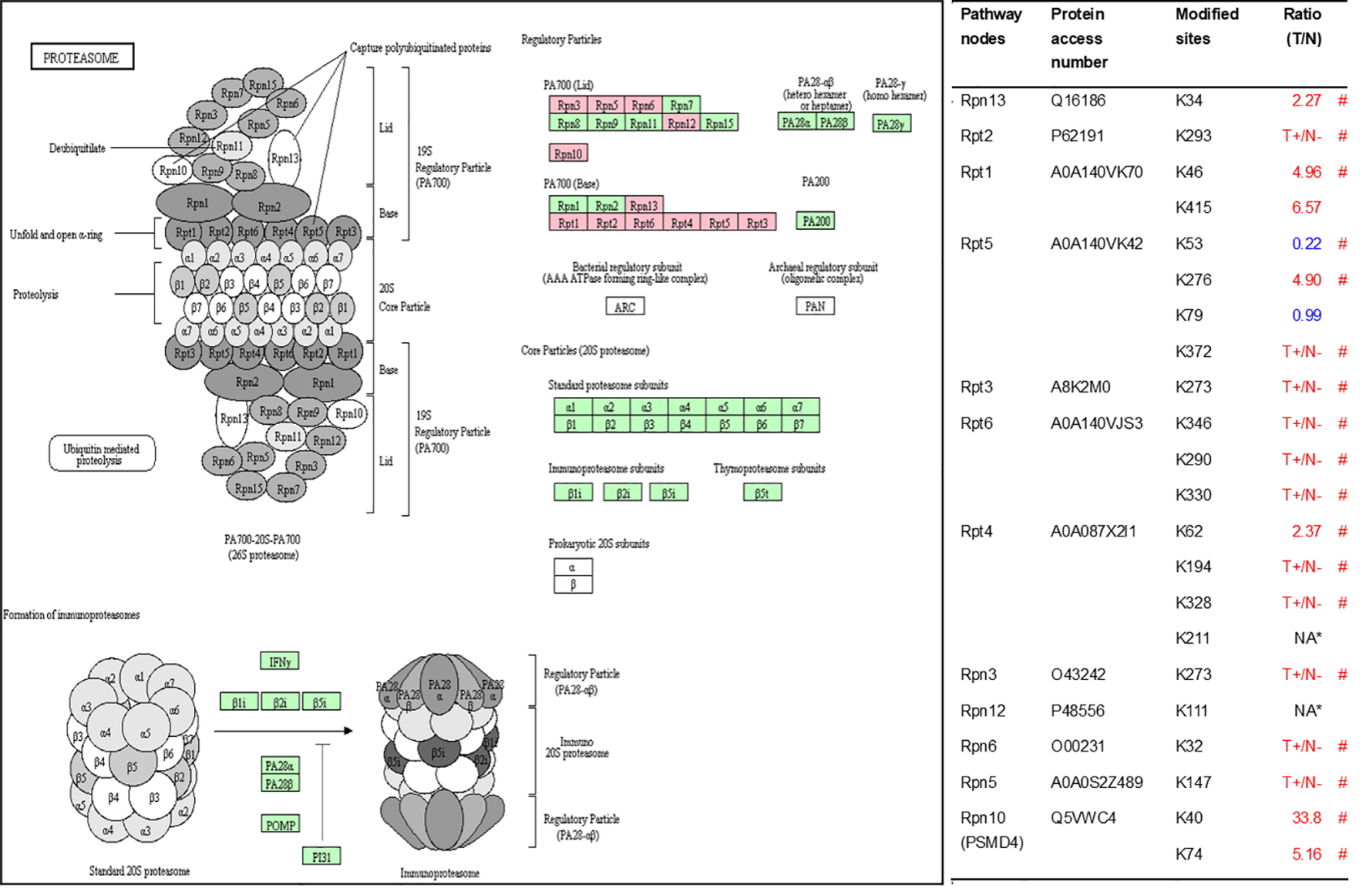
Ubiquitination-mediated proteasome pathway. Pink=the identified ubiquitinated subunits. Green = non-ubiquitinated subunits. K* = ubiquitinated lysine residue. Red indicates the degree of ubiquitination of this site is upregulated, and blue indicates downregulated. Ratio(T/N) = Ratio of tumor to control. T+/N- = ubiquitination only in tumor. NA* means no quantitative information in both tumor and control. # means statistically significantly altered ubiquitination level in tumor compared to controls.

As described in **Table 1**, multiple proteins are decreased or increased in LSCC tissues relative to control tissues, which might be due to the alteration of ubiquitination-mediated protein synthesis-degradation system. (i) Ribosome, the central site for protein synthesis or translation, is a dense ribonucleoprotein particle that includes two subunits (large and small), and each subunit contains one rRNA and many different protein molecules. The small subunit is responsible for sequence-specific recognition of the template mRNA, such as the recognition of the initial part, the interaction of codon with anti-codon, and the binding site of mRNA is also on this subunit. The large subunit is responsible for carrying amino acids and tRNAs, including peptide bond formation, and peptidyl-RAN binding. In this study, 7 UPs [ribosomal proteins L3e (P39023), L11e (P62913), L9e (P32969), L7Ae (P62424), L40e (P62987), L18Ae (Q02543), and L12e (A0A024RBS2)] were identified in large subunit with significantly increased ubiquitination level in ribosomal proteins L11e, L12e, L18Ae, and L40e in LSCC tissues relative to controls, and 6 UPs [ribosomal proteins S20e (P60866), S3e (P23396), S2e (P15880), S16e (P62249), S27Ae (B2RDW1), and S9e (A0A024R4M0)] in small subunit with significantly increased ubiquitination level in ribosomal proteins S3e, S9e, S16e, and S27e in LSCC tissues relative to controls (**Figure 7**). (ii) Endoplasmic reticulum (ER) is the cellular organelle, and is responsible for the assembly of multi-subunit proteins, formation of proper conformation of protein, protein secretion, lipid biosynthesis, and calcium homeostasis. The correctly folded proteins are transited into the Golgi complex. ER stress occurs under normal or pathophysiological conditions, which can lead to accumulation of misfolded proteins in the ER lumen to restore the correct fold. Proteins that ultimately failed to recover to normal folding were transported to the proteasome for ubiquitination-mediated degradation, which is named as ER-associated degradation (ERAD) (31). Accumulation of misfolded proteins can also lead to ER stress and ultimately lead to the activation of a range of signaling pathways, which was called UPR (unfolded protein response). In the case of severely damaged cells, UPR is insufficient to restore the function of ER, when the cells undergo apoptosis. In this study, 13 UPs [EIF2AK2 (P19525), CAPN1 (P07384), BCAP31 (P51572), EL52 (K9JA46), YOD1 (Q5VVQ6), UBE4B (O95155), RAD23B (P54727), NGLY1 (Q96IV0), VCP (P55072), HSPA8 (P11142), DNAJB1 (P25685), HSP90AB1 (A0A024RD80), and RAD23A (A0A024R7G8)] with 39 ubiquitination sites were identified in protein ER processing pathway, wth significantly decreased ubiquitination levels in proteins YOD1, and VCP (K8, K18, K109, K389, K651, K658, and K668), and with significantly increased ubiquitination levels in proteins BCAP31, EL52, RAD23B, VCP(K217, and K614), HSPA8, and DNAJB1 (**Figure 8**). (iii)Ubiquitination was multiple enzymatic reaction process that requires enzymes E1, E2, and E3. In this study, 9 Ups, including six E3s [UBE4B (O95155), CUL5 (Q93034), ITCH (Q96J02), CUL4A (Q13619), PML (P29590), and HUWE1 (A0A024R9W5)], two E2s [UBE2O (Q9C0C9), and UBE2N (P61088)], and one E1 [UBE1 (A0A024RDB0)], were identified, with significantly decreased ubiquitination levels in E2 UBE2N, and with significantly increased ubiquitination levels at E3s ITCH, HUWE1, and CUL4A, in LSCC tissues relative to controls (**Figure 9**). For these six E3s, HUWE1 and ITCH were HECT type E3, UBE4B were U-box type E3, PML was single RING-finger type E3, and CUL5 and CUL4A were multi subunit RING-finger type E3. (iv) Proteasome is the crucial machine to response for ubiquitin-mediated proteolysis, which includes one 20S core, one 19S regulatory lid (PA700), and one 19S regulatory base (PA700) in the 26S proteasome complex. The 20S core particle provides an enclosed cavity where proteins are degraded. This present study identified 7 UPs (Rpn13, and Rpt 1-6) in PA700 (Base) with significantly increased ubiquitination levels in proteins Rpn13, Rpt1, Rpt3, Rpt4, Rpt5 (K276 and K372), and Rpt6, and significantly decreased ubiquitination level in protein Rpt 5 (K53); and 5 UPs (Rpn 3, 5, 6, 10, and 12) in PA700 (Lid) with significantly increased ubiquitination level, in human LSCC tissues relative to controls (**Figure 10**), but no any ubiquitination was found in the 20S core.

**Table 1:** Differentially expressed proteins with significantly ubiquitination alterations in LSCC tissues compared to controls.

Accession No.	Gene name	Protein name	Ubiquitinated peptides	Ubiquitinated positions	Differentially ubiquitinated Sites Ratio (T/N)	Differentially expressed protein Ratio (T/N)
A0A024R1N1	MYH9	Myosin, heavy polypeptide 9, non-muscle, isoform CRA_a	DYVQK*AQTK	403	6.391	1.66
			VK*PLLQVSR	835	8.094	
			VAAYDKLEK*TK	1413	T+/N-	
			K*AGKLDPHLVLDQLR	679	T+/N-	
A0A024R3E3	APOA1	Apolipoprotein A-I, isoform CRA_a	DYVSQFEGSALGK*QLNLK	64	0.039	4.80
			VSFLSALEEYTK*K*	263	T-/N+	
			VSFLSALEEYTK*K	262	T-/N+	
			VQPYLDDFQKK*	131	T-/N+	
A0A024RAY2	KRT18	Keratin 18, isoform CRA_a	NLK*ASLENSLR	317	9.286	0.52
A0A087WUZ3		Spectrin, beta, erythrocytic (Includes spherocytosis, clinical type I) variant (Fragment)	IHCLENVDK*ALQFLK	118	T-/N+	0.68
A0A0D9SGC1	MYO6	Unconventional myosin-VI	SLDSYPVTSK*NDGTRPK	1045	T-/N+	0.24
A0A0G2JIW1	HSPA1B	Heat shock 70 kDa protein 1B	LIGDAAK*NQVALNPQNTVFDAK	57	2.302	0.62
			AMTK*DNNLLGR	452	3.120	
			MVQEAEK*YKAEDEVQR	525	3.486	
			YK*AEDEVQR	527	3.554	
			RK*ELEQVCNPIISGLYQGAGGPGPGGFGAQGPK*	598	8.518	
			QATK*DAGVIAGLNVLR	160	10.868	
			ANK*ITITNDK	501	14.562	
			K*FGDPVVQSDMK	78	T+/N-	
			VLDK*CQEVISWLDANTLAEKDEFEHK	574	T+/N-	
A0A0S2Z3S6	CYBB	Cytochrome b-245 beta polypeptide isoform 1 (Fragment)	VVITK*VVTHPFK	299	T+/N-	1.64
A8K287	SNAP23	Synaptosomal-associated protein	TITMLDEQK*EQLNR	49	T+/N-	0.60
B2R5T5	PRKAR1A	Protein kinase, cAMP-dependent, regulatory, type I, alpha (Tissue specific extinguisher 1), isoform CRA_a	VSILESLDK*WER	261	0.086	0.43
B2R6J2	EZR	Ezrin	FGDYNK*EVHK	139	0.151	0.33
D9YZU5	HBD	Delta globin	K*VLGAFSDGLAHLDNLK	67	0.032	1.42
			VHLTPEEK*SAVTALWGK	9	0.052	
			FFESFGDLSTPDAVMGNPKVK*	62	0.056	
			GTFATLSELHCDK*LHVDPENFR	96	0.096	
			VVAGVANALAHK*YH	145	0.165	
			VLGAFSDGLAHLDNLK*GTFATLSELHCDK	83	T-/N+	
			FFESFGDLSTPDAVMGNPK*VK	60	T-/N+	
G3V1N2	HBA2	HCG1745306, isoform CRA_a	TYFPHFDLSHGSAQVK*GHGK	25	0.045	0.64
			MFLSFPTTK*TYFPHFDLSHGSAQVK	9	0.142	
O95864	FADS2	Fatty acid desaturase 2	EVSVPTFSWEEIQK*HNLR	28	28.124	0.65
P00915	CA1	Carbonic anhydrase 1	HDTSLK*PISVSYNPATAK	46	0.078	7.70
			TSETK*HDTSLKPISVSYNPATAK	40	T-/N+	
			ASPDWGYDDK*NGPEQWSK	11	T-/N+	
P04075	ALDOC	Fructose-bisphosphate aldolase	YASICQQNGIVPIVEPEILPDGDHDLK*R	200	3.801	1.60
P04075	ALDOA	Fructose-bisphosphate aldolase A	VDK*GVVPLAGTNGETTTQGLDGLSER	111	3.837	0.63
P04083	ANXA1	Annexin A1	AAMK*GLGTDEDTLIEILASR	128	0.388	1.58
			CATSK*PAFFAEK	274	6.312	
			DLAK*DITSDTSGDFR	166	T+/N-	
P04406	GAPDH	Glyceraldehyde-3-phosphate dehydrogenase	TVDGPSGK*LWR	194	2.669	6.10
			GALQNIIPASTGAAK*AVGK	215	3.215	
			VVK*QASEGPLK	263	3.450	
			VIHDNFGIVEGLMTTVHAITATQK*TVDGPSGK	186	25.358	
			AGAHLQGGAK*R	117	T+/N-	
P04792	HSPB1	Heat shock protein beta-1	DGVVEITGK*HEER	123	30.905	5.80
			AQLGGPEAAK*SDETAAK	198	T+/N-	
P04899	GNAS	Guanine nucleotide-binding protein G(s) subunit alpha isoforms Xlas	LLLLGAGESGK*STIVK	46	T+/N-	9.50
P06454	PTMA	Prothymosin alpha	SDAAVDTSSEITTK*DLK	15	T+/N-	5.60
P06576	ATP5B	ATP synthase subunit beta, mitochondrial	VLDSGAPIK*IPVGPETLGR	133	T+/N-	2.68
P06702	S100A9	Protein S100-A9	TCK*MSQLER	4	0.172	3.18
			LGHPDTLNQGEFK*ELVR	38	T+/N-	
P08069	IGF1R	Insulin-like growth factor 1 receptor	VAIK*TVNEAASMR	1033	T+/N-	5.50
P08670	VIM	Vimentin	QQYESVAAK*NLQEAEEWYK	282	0.129	2.40
			K*VESLQEEIAFLK	223	0.169	
			SK*FADLSEAANR	294	0.204	
			RQVDQLTNDK*AR	168	0.210	
			TLLIK*TVETR	445	0.258	
			FLEQQNK*ILLAELEQLKGQGK	129	0.293	
			ETNLDSLPLVDTHSK*R	439	0.361	
			RQVQSLTCEVDALK*GTNESLER	334	0.363	
			LREK*LQEEMLQR	188	0.406	
			ILLAELEQLK*GQGK	139	0.491	
P08670	DES	Mutant desmin	K*LLEGEESR	402	0.264	0.50
P09936	UCHL1	Ubiquitin carboxyl-terminal hydrolase isozyme L1	MQLK*PMEINPEMLNK	4	16.321	0.58
			CFEK*NEAIQAAHDAVAQEGQCR	135	T+/N-	
P14735	IDE	Insulin-degrading enzyme	EVNAVDSEHEK*NVMNDAWR	192	0.039	1.86
P16070	CD44	CD44 antigen	SQEMVHLVNK*ESSETPDQFMTADETR	715	4.018	6.80
P16152	CBR1	Carbonyl reductase [NADPH] 1	ALK*SCSPELQQK	148	T+/N-	5.80
P20700	LMNB1	Lamin-B1	K*QLADETLLK	182	T+/N-	0.47
			IESLSSQLSNLQK*ESR	312	T+/N-	
P27708	CAD	CAD protein	NK*SELLPTVR	1325	7.916	1.73
			DDQLK*VIECNVR	1211	T+/N-	
			LSSFVTK*GYR	1411	T+/N-	
			LGPGK*GEVRPELGSR	1657	T+/N-	
P29353	SHC1	SHC-transforming protein 1	DLFDMK*PFEDALR	462	T+/N-	1.79
P31946	YWHAB	14-3-3 protein beta/alpha	VISSIEQK*TER	70	0.045	0.60
P46734	MAP2K3	Dual specificity mitogen-activated protein kinase kinase 3	ATVNSQEQK*R	105	T+/N-	0.74
P48507	GCLM	Glutamate–cysteine ligase regulatory subunit	EFPDVLECTVSHAVEK*INPDER	80	T+/N-	2.61
P62249	RPS16	40S ribosomal protein S16	LLEPVLLLGK*ER	60	T+/N-	0.47
P63218	GNG5	Guanine nucleotide-binding protein G(I)/G(S)/G(O) subunit gamma-5	SGSSSVAAMKK*	12	T-/N+	4.70
P67936	TPM4	Tropomyosin alpha-4 chain	AGLNSLEAVK*R	11	T+/N-	3.00
P68104	EEF1A1	Elongation factor 1-alpha 1	AAGAGK*VTK	450	26.878	0.61
			QTVAVGVIK*AVDKK	439	108.563	
			QLIVGVNK*MDSTEPPYSQK	154	T+/N-	
			FEK*EAAEMGK	44	T+/N-	
			VETGVLK*PGMVVTFAPVNVTTEVK	273	T+/N-	
			KLEDGPK*FLK	392	T+/N-	
P98172	EFNB1	Ephrin-B1	AAALSLSTLASPK*GGSGTAGTEPSDIIIPLR	289	T+/N-	2.38
Q12965	MYO1E	Unconventional myosin-Ie	DIILQSNPLLEAFGNAK*TVR	160	T+/N-	0.61
Q4W4Y1	DRIP4	Dopamine receptor interacting protein 4	MK*QSNNEANLR	640	2.767	1.49
Q6IA69	NADSYN1	Glutamine-dependent NAD(+) synthetase	HK*MTTLTPAYHAENYSPEDNR	649	T+/N-	4.20
Q6IBN1	HNRPK	HNRPK protein	IILDLISESPIK*GR	219	85.542	4.70
			HESGASIK*IDEPLEGSEDR	422	T-/N+	
			ILLQSK*NAGAVIGK	52	T+/N-	
			LLIHQSLAGGIIGVK*GAK	163	T+/N-	

The symbol * means K* = ubiquitinated lysine residue. T+/N- means that ubiquitination occurred in tumors but not in controls. T-/N+ means that ubiquitination occurred in controls but not in tumors.

### Overlapping analysis of differentially ubiquitinated proteins and differentially expressed proteins in LSCC

One of the consequences of ubiquitination is to degrade proteins. Those DEPs with differentially ubiquitinated modifications might have more relevance to tumorigenesis. A set of 265 DEP data in LSCC tissues compared to controls extracting from the published data (32–36) (**Supplementary Table 12**) were compared to this set of 464 DUP data with 789 differentially ubiquitinated sites in LSCC tissues compared to controls (**Supplementary Table 13**). A total of 45 DEPs in LSCC were found to have significantly differentially ubiquitinated modifications (**Table 1**). When one analyzed the relationship between DUP and DEP in LSCC, those 45 DEPs were grouped into 5 categories (**Table 2**): (i) When the ubiquitination level was decreased, the protein expression level was increased, including APOA1, HBD, CA1, VIM, IDE, and GNG5; (ii) When the ubiquitination level was increased, the protein expression level was decreased, including KRT18, HSPA1B, SNAP23, FADS2, ALDOA, UCHL1, LMNB1, MAP2K3, RPS16, EEF1A1, and MYO1E; (iii) When the ubiquitination level was decreased, the protein expression level was decreased, including MYO6, PRKAR1A, EZR, HBA2, DES, YWHAB, and erythrocytic spectrin beta; (iv) When the ubiquitination level was increased, the protein expression level was increased, including MYH9, CYBB, ALDOC, GAPDH, HSPB1, GNAS, PTMA, ATP5B, IGF1R, CD44, CBR1, CAD, SHC1, GCLM, TMP4, EFNB1, DRIP4, and NADSYN1; and (v) when a protein existed both increased and descresed ubiquitination sites, the protein expression level was increased, including ANXA1, S100A9, and HNRPK.

**Table 2:** The relationship between DUP and DEP in LSCC tissues compared to controls.

DUP	DEP	Proteins
–	+	APOA1, HBD, CA1, VIM, IDE, GNG5
+	–	KRT18, HSPA1B, SNAP23, FADS2, ALDOA, UCHL1, LMNB1, MAP2K3, RPS16, EEF1A1, MYO1E
–	–	MYO6, PRKAR1A, EZR, HBA2, DES, YWHAB, Erythrocytic spectrin beta
+	+	MYH9, CYBB, ALDOC, GAPDH, HSPB1, GNAS, PTMA, ATP5B, IGF1R, CD44, CBR1, CAD, SHC1, GCLM, TMP4, EFNB1, DRIP4, NADSYN1
+/-	+	ANXA1, S100A9, HNRPK

For DUP, - means the significantly decreased ubiquitination level; + means the significantly increased ubiquitination level; and +/- means that the significantly increased and decreased ubiqutination sites existed in a protein. For DEP, + means upregulation; and – means downregulation.

## Discussion

### Protein ubiquitination profile and ubiquitination-mediated signaling pathways in LSCC

To investigate ubiquitination profile and its potential functions in LSCC, and to promote PPPM of LSCC, label-free quantitative proteomics was used to investigate ubiquitination in LSCC tissues. This present study provided the first comprehensive, quantitative ubiquitination profile in LSCC tissues. A total of 627 UPs with 1209 ubiquitination sites were identified, and 93.7% of identified ubiquitination sites had quantitative intensity. These UPs were involved in multiple functional categories, cellular biological processes, and signaling pathways in LSCC. In total, 47 statistically significant pathways were identified to involve UPs in LSCC, and most of these signaling pathways were cancer-related pathways, including ubiquitin-mediated proteolysis, ribosome complex, ER-mediated protein processing, proteasome complex, biosynthesis of amino acids, PI3K-Akt signaling pathway, Ras signaling pathway, mTOR signaling pathway, HIF-1 signaling pathway, cell cycle, apoptosis pathway, glycolysis/gluconeogenesis, carbon metabolism, central carbon metabolism in cancer, fructose and mannose metabolism, metabolomic pathways, drug metabolism-other enzymes, sphingolipid signaling pathway, tight junction, adherens junction, gap junction, non-homologous end-joining, endocytosis, SNARE interactions in vesicular transport, dopaminergic synapse, GABAergic synapse, chemokine signaling pathway, Rap1 signaling pathway, proteoglycans in cancer, pathways in cancer, microRNAs in cancer, leukocyte transendothelial migration, regulation of actin cytoskeleton, and platelet activation. These ubiquitinaiton-mediated signaling pathways provide the functional profiles of ubiquitination in LSCC.

### Ubiquitination-mediated tumor protein turnover in LSCC

It is well-known that one of main functions of ubiquitination is involved in protein degradation, and participates in protein turnover in tumor pathophysiological processes. Interestingly, this study found that ubiquitination was significantly involved in four protein turnover-associated pathways, including ribosome complex, ubiquitin-mediated proteolysis, ER-mediated protein processing, and proteasome complex pathways, which are discussed in detailed here.

Proteins are vital parts of living organisms, and are in dynamic balance between generation and degradation. Protein turnover is the protein synthesis-degradation balance, which gives cells the flexibility to adjust the abundance and function of the protein in response to various intracellular or extracellular stimuli (37). Protein synthesis is determined by both transcription and translation. The mRNA transcribed into the cytoplasm and binds to the ribosome to begin the process of protein synthesis. The overall activity of ribosome and the rate of translation initiation and elongation have an important effect on the rate of protein synthesis. The newly synthesized protein does not have a spatial structure and corresponding functions, which needs to be folded and assembled in ER. The processing time of different proteins in ER is also different. The UPS and autophagy are the main pathways for protein degradation (38, 39). The former selectively degrades the misfolded proteins and short half-life proteins that have been labeled with ubiquitin in the proteasome, while the latter transports denatured long-lived proteins and damaged or excess organelles to the lysosome for degradation. The ability to adjust protein abundances in a timely and precise manner based on external environmental stimuli is critical to maintain the normal function of cells. Protein turnover abnormalities are associated with the development of multiple diseases, including neurodegenerative diseases (40, 41) and tumors (42, 43). Targeting altered protein turnover provides new opportunities and challenges to develop anti-tumor drugs (44).

Studies demonstrate that multiple ribosomal subunits have been ubiquitinated in eukaryotes (45–47). This study also found 13 ubiquitinated ribosome subunits in LSCC, which demonstrates the important regulatory roles of ubiquitination in ribosomal complex. Ubiquitination affects protein turnover by affecting the function of ribosomes in several ways.

First, ubiquitination regulates multiple steps of ribosome biogenesis (48), which involves the production and correct assembly of rRNAs and ribosomal proteins. Abnormal ribosome biogenesis inevitably affects the turnover of intracellular proteins. However, this study did not find the ribosome biogenesis pathway in KEGG enrichment analysis, which suggests that ubiquitination might not affect protein turnover by participating in regulating ribosome biogenesis in LSCC. Moreover, ubiquitination of ribosomal proteins regulates ribosome-associated quality control (RQC) (49, 50). Ribosomes are not only responsible for protein synthesis, but also execute RQC to minimize production of aberrant proteins. Many factors cause abnormal protein synthesis in ribosomes. RQC is a biologically evolved surveillance mechanism that timely terminates the protein synthesis of ribosomes, and initiates corresponding pathways to degrade nascent polypeptides when there exist interrupted translation (51). Obviously, dysfunction of RQC results in accumulating abnormal proteins in cells, which eventually affects the protein turnover. Study showed that the poly (A) tails of mature mRNA stalled ribosomes, repressed downstream translation, and initiated RQC; this is a process known as ribosome stall resolution (49). One study showed that in the initiation of this process, it is essential for ubiquitin ligase ZNF598 to catalyze ubiquitination at residue K_8_ in RPS20 (P60866) and at residues K_138_ and K_139_ in RPS10, and the ubiquitination failure of RPS20 or RPS10 would lead to defective resolution of stalled ribosomes (50). Another study also found that ZNF598 primarily mono-ubiquitinated two lysine residues K_4_ and K_8_ in RPS20, which was required to stall ribosomes during poly (A) translation (49). In the yeast model, study found that ubiquitination at residue K_8_ in 40S ribosomal protein uS10 (corresponding to RPS20 in human) catalyzed by E3 ubiquitin ligase Hel2 switched on RQC (52). Those studies showed that ubiquitination at residue K_8_ in RPS20 was species conservation, and played a pivotal role in RQC pathways. Besides RPS20, recent study demonstrated that the balance between mono-ubiquitination at residue K_214_ in RPS3 (P23396) catalyzed by RNF123 E3 ligase and deubiquitination by USP10, also participated in the regulation of RQC (53). This study found ubiquitination level was decreased at residue K_8_ (T/N ratio=0.95, *P* value = 0.78) in RPS20 and increased at residue K_214_ (T/N ratio=5.03, *P* value = 0.32) in RPS3, which means that the impaired RQC in LSCC tissue may contribute to the abnormal protein turnover. Further, ribosome ubiquitination participated in reprogramming of ribosomal protein translation that was induced by unfolded protein reaction (UPR). UPR not only induced ER stress and disturbed protein homeostasis, but also reprogramed translation; and persistent UPR might lead to cell death. Reprogramming of ribosomal protein translation is an intracellular response to protein homeostasis, and it can also affect the turnover of intracellular proteins. One study demonstrated that ubiquitination at residues K_58_ or K_275_ in RPS2 (P15880) and at residue K_8_ in RPS20 participate in UPR-induced ribosomal translation reprogram, and these two proteins without ubiquitination at these residues promoted UPR-induced cell death (54). This study identified ubiquitination at residues K_58_ (T/N = 1.4; *P* value = 0.06) and K_275_ (T/N = 2.21; *P* value = 0.21) in RPS2 increased in LSCC. Therefore, ubiquitination in proteins RPS3, RPS20, and RPS2 in 40S subunits play an important role in ribosome-related protein turnover regulation.

Both ribosome and ER are indispensable organelles for intracellular protein homeostasis and protein turnover, and this study demonstrates that ubiquitination also has regulatory effects on ER. Among the various biological processes of ER, UPR and ERAD are essential for maintaining protein homeostasis and turnover. The former regulates the expression of multiple downstream genes through at least three pathways to recover UPR-induced ER stress (55). The latter relieves ER stress through the process of retro-translocation (terminally misfolded proteins were translocated from the ER lumen into the cytoplasm through the translocation pore in the ER membrane) and ultimately degraded by proteasome (56). Our results showed that UPs mainly concentrated in the above two biological processes. For example, study demonstrated that EIF2AK2 (P19525) was responsible for starting a branch of the UPR. When ER stress was activated, EIF2AK2 itself was oligomerized and phosphorylated, and also ubiquitously translational initiation factor eIF2a was indirectly inactivated to inhibit translation of mRNA. Thus, EIF2AK2 inhibits the flux of protein to enter ER to alleviate ER stress and restore protein homeostasis (55). This study qualitatively identified the ubiquitination at residue K_408_ in EIF2AK2. Currently, the exact effect of ubiquitination on the function of EIF2AK2 is not clear, but study reported that the residues K_69_ and K_159_ have been ISGylated, one type of ubiquitin-like modification, that down-regulated protein translation (57). During ERAD, studies showed that molecular chaperones not only assist in folding of the protein in ER, but also play a variety of important functions to ensure ERAD (58). These molecular chaperones assist degradation through substrate recognition and preventing substrate aggregation (59). This study identified the ubiquitination of three molecular chaperones (HSPA8, DNAJB1 and K9JA46) indicating that ubiquitination can affect the function of molecular chaperones, which in turn affects ERAD and protein turnover.

The UPS is another determinant of intracellular protein turnover, because the degradation of many proteins is carried out by UPS in mammalian cells (60). Under physiological conditions, the activity of UPS is dynamically regulated by different signaling pathways in response to various stimuli (60). As mentioned above, UPS contains a large number of components, so dysfunction of any one component can lead to abnormal protein degradation, which eventually affects protein turnover. In this study, it was found that 11 proteasome subunits (all belong to the 19S regulatory subunit) and 9 UPS-related enzymes were ubiquitinated, which revealed that ubiquitination can regulate protein turnover by regulating proteasome activity and UPS-related enzymes.

The 26S proteasome is a core component of UPS, and proteins labeled with K48-linked polyubiquitin chains are recognized by its 19S regulatory subunit (RS) and degraded by its 20S core subunit (CS) (61, 62). A variety of factors affect proteasome activity, such as proteasome biogenesis and PTMs (63). For example, the RS has two subunits (lid an base), among which the base assembly is associated with 4 RS assembly chaperones (RACs): Rpn14 (PAAF1), S5b (PSMD5), p28 (PSMD10), and p27 (PSMD9) (64). Loss of these chaperones leads to base subcomplex of RS assembly defect (65). This study identified ubiquitination at residues K_23_ (no quantitative information) and K_30_ (T+/N-) in PSMD10 (O75832) in LSCC for the first time. We hypothesize that ubiquitination may affect the intracellular abundance or function of PSMD10, which finally affects proteasome biogenesis and the activity of proteasome. The specific regulatory mechanism needs further research. PTMs of various proteasome subunits make the regulation of proteasome function more complicated. Ubiquitination is one of the 11 PTMs of proteasome subunits that have been identified so far (66). At present, phosphorylation has been deeply studied in the regulation mechanism of proteasome activity, and researches had revealed that a variety of kinases and phosphatases regulate proteasome (67). The functional consequences of other PTMs (including ubiquitination) in regulation of proteasome are presently lacking. In our study, all ubiquitinated proteasome subunits belong to the RS, which suggests that ubiquitination may affect the activity of the proteasome by regulating the function of the RS not the CS in LSCC. Under normal circumstances, Rpn13 and Rpn10/S5a are responsible for identification of the ubiquitin chains on the protein committed to degradation, after that protein is translocated through the ATPase ring into the CS (68). However, when the proteasome is impaired under various circumstances (such as proteotoxic stresses and treated with bortezomib), Ube3c ubiquitinates residues K_21_ and K_34_ in Rpn13 and inhibits the binding of 26S particles to ubiquitin conjugates (69). Our study found the increased ubiquitination level at residue K_34_ (T/N=2.27; *p* = 0.01) in Rpn13, which can be seen as a sign of impaired proteasome function in LSCC. More importantly, if we can confirm the above results in LSCC, the degree of ubiquitination of Rpn13 can be used as a biomarker to predict the severity of impaired proteasome function, and the biological effects produced by proteasome activity inhibitors *in vivo*. In addition to the ubiquitination of Rpn13, monoubiquitination of Rpn10 induced by Rsp5, a member of NEDD4 ubiquitin protein ligase family, significantly inhibits the interaction of Rpn10 with substrates to decrease the activities of proteasome complex (70). Different from the previous yeast-based research that discovered four ubiquitination sites (K_71_, K_84_, K_99_, and K_268_), this study identified significantly increased ubiquitination levels at residues K_40_ (N/T = 33.8; *p* < 0.01) and K_74_ (N/T = 5.2, p = 0.01) in Rpn10 in LSCC tissue. Both K_40_ and K_74_ in Rpn10 are located in the VWFA domain that has been implicated to be responsible for stabilizing the lid-base association of RS. In LSCC, monoubiquitination of K_40_ and K_74_ in Rpn10 may participate in the regulation of proteasome activity.

In addition to proteasome biogenesis and PTMs, the amount of some proteasome subunits can also affect the activity of proteasome. For example, the activity of the proteasome is positively correlated with the expression level of PSMD11, and overexpression of PSMD11 increases the activity of the proteasome in stem cells (71). Specially, the dysregulated proteasomal activities were showed in the muscles of PSMC4-knockout animals (72). The ubiquitination of both PSMD11 and PSMC4 have been identified in this study. If these proteins are degraded by UPS, which means ubiquitination of these proteins will affect the activity of proteasome. So it is especially important to clarify the specific ubiquitination regulatory mechanisms of these proteins. As mentioned above, few studies are involved in the impact of ubiquitination on proteasome subunits, ubiquitination proteomics can be used as a powerful screening tool to provide direction for further studies.

Above description discussed the effect of ubiquitination on proteasome activity. Further, we discuss the ubiquitination regulation on the UPS-related enzymes. Our study found 9 UPS-related enzymes including E1, E2 and E3, which indicate that enzymes in the process of protein ubiquitination are also regulated by ubiquitination. Currently, studies have focused on the roles of ubiquitin in these enzymes; however, just relatively few studies were focused on the roles of the ubiquitination in these 9 enzymes. For instance, PML (P29590) was ubiquitinated by several E3s to result in the degradation of proteasome complex (73, 74). ITCH (Q96J02) can be ubiquitinated by itself *via* lysine-63 linkages to control the cytoplasmic-nuclear shuffling of ITCH (75). Of course, ubiquitination and even other PTMs might extensively affect these enzyme activities, currently known ubiquitination are only the tip of the iceberg.

### The relationship between ubiquitination and protein expression level in LSCC

One of main functions of ubiquitination is to degrade proteins. A total of 265 DEP data in LSCC tissues was obtained from the published data (32–36) (**Supplementary Table 12**). This study identified 464 DUP data with 789 differentially ubiquitinated sites in LSCC tissues compared to controls (**Supplementary Table 13**). Overlapping analysis of the DUP data and DEP data found 45 DEPs with significantly altered ubiquitination level (**Table 1**). Five types of relationships between DUP and DEP were found in LSCC (**Table 2**), including: (i) the protein expression level was increased with the decrease of ubiquitination level, which might be due to the constant synthesis velocity and the decreased degradation velocity; (ii) the protein expression level was decreased with the increase of ubiquitination level, which might be due to the constant synthesis velocity and the increased degradation velocity; (iii) the protein expression level was decreased with decreased of the ubiquitination level, which might be because the protein degradation velocity was faster than its synthesis velocity; (iv) the protein expression level was increased with the increase of the ubiquitination level, which might be because protein degradation velocity was slower its synthesis velocity; (v) the protein expression level was increased with both increase and decrease of ubiquitination sites in a protein, this type of reseason was complex. These results clearly demonstrate the complexity of protein synthesis-degradation system. Further experiment studies would be needed to confirm the real status. Moreover, one should note that only 265 DEPs are obtained due to a limited list of DEPs in the published literature, an expanded quantitative proteomics would be needed to identify more DEPs in LSCC tissues compared to controls, which will reveal more DEPs with significant ubiquitination alteration.

## Conclusions

This study provides the first quantitative ubiquitination proteomics analysis of LSCC tissue, and shows that ubiquitination can affect the occurrence and development of LSCC in multiple biological functions and pathways. Here focuses on the regulation mechanism of ubiquitination on intracellular protein turnover-related pathways. The results showed that ubiquitination affected UPS functions by regulating proteasome activity and UPS-related enzymes. In addition, ubiquitination also affected protein turnover by regulating ribosome assembly, ribosome-associated quality control (RQC), ER stress response, and ER-associated degradation (ERAD). Although ubiquitination studies in tumors have made significant progress, many anti-tumor drugs have been clinically applied and have achieved excellent therapeutic effects, yet considering that ubiquitination has extensive regulatory networks and the complexity of ubiquitination itself, so there is still a lot of unknown things waiting for ones to explore. This study paved the way for further exploration of the specific regulatory mechanisms of ubiquitination in LSCC.

## Data availability statement

The datasets presented in this study can be found in online repositories. The names of the repository/repositories and accession number(s) can be found in the article/**Supplementary Material**.

## Ethics statement

The studies involving human participants were reviewed and approved by Xiangya Hospital Medical Ethics Committee of Central South University, China. The patients/participants provided their written informed consent to participate in this study.

## Author contributions

XHZ conceived the concept, designed experiments and manuscript, instructed experiments and data analysis, supervised results, coordinated, wrote and critically revised manuscript, and was responsible for its financial supports and the corresponding works. ML collected tissue samples, analyzed ubiquitinomic data, prepared figures and tables, and wrote the manuscript. XZ and LY analyzed the relationship between ubiquitination and protein differential expression. JY, XHZ, SZ, YG, BL, SW, JL, and NL participated in partial experiments. All authors approved the final manuscript.

## Funding

This work was supported by the Shandong First Medical University Talent Introduction Funds (to XZ) , Shandong First Medical University High-level Scientific Research Achievement Cultivation Funding Program (to XZ), the Shandong Provincial Natural Science Foundation (ZR202103020356 or ZR2021MH156 to XZ), and the Academic Promotion Program of Shandong First Medical University (2019ZL002).

## Conflict of interest

The authors declare that the research was conducted in the absence of any commercial or financial relationships that could be construed as a potential conflict of interest.

## Publisher’s note

All claims expressed in this article are solely those of the authors and do not necessarily represent those of their affiliated organizations, or those of the publisher, the editors and the reviewers. Any product that may be evaluated in this article, or claim that may be made by its manufacturer, is not guaranteed or endorsed by the publisher.

## Abbreviations

ALK, anaplastic lymphoma kinase; AUC, area under the curve; BP, biological processes; CC, cellular components; CS, core subunit; DTT, dithiothreitol; E1, ubiquitin-activating enzyme; E2, ubiquitin-conjugating enzyme; E3, ubiquitin ligase; EPC, edge percolated component; ER, endoplasmic reticulum; GO, gene ontology; IAP, immunoaffinity purification; ICAT, isotope-coded affinity tags; iTRAQ, isobaric tags for relative and absolute quantification; KEGG, Kyoto Encyclopedia of Genes and Genomes; LC, liquid chromatography; LSCC, lung squamous cell cancer; MCC, maximal clique centrality; MCODE, molecular complex detection; MF, molecular functions; MNC, maximum neighborhood component; MS/MS, tandem mass spectrometry; NSCLC, non-small-cell lung cancer; PMSF, phenylmethyl-sulfonyl fluoride; PPI, protein-protein interaction; PPPM, predictive, preventive and personalized medicine; PTM, post-translational modification; RACs, RS assembly chaperones; RQC, ribosome-associated quality control; RS, regulatory subunit; SILAC, isotope labels with amino acids in cell culture; TAC, tumor-adjacent control; TFA, Trifluoroacetic acid; TKI, tyrosine kinase inhibitors; TTP, time to progression; UPs, ubiquitinated proteins; 2D-DIGE, 2D fluorescence difference in-gel electrophoresis; 2D-PAGE, two-dimensional polyacrylamide gel electrophoresis.
