# Supplementary material for: Ubiquitination-mediated molecular pathway alterations in human lung squamous cell carcinomas identified by quantitative ubiquitinomics
Source: Front Endocrinol (Lausanne). 2022 Sep 15;13:970843. doi: 10.3389/fendo.2022.970843 (PMC9520991; doi:10.3389/fendo.2022.970843)
Supplement: Supplementary file 1 [file DataSheet_1.zip › Supplemental Table 1_v6.doc]

**Supplemental Table 1. Pathological diagnosis of LSCC and its adjacent control tissues.** LSCC = lung squamous cell carcinoma. F = female. M = male. These samples were at the early stage of LSCC.

| **Sample ID** | **Gender** | **Age (year)** | **Smoking (Year)** | **Diagnosis; location** | **Tissues** |
| --- | --- | --- | --- | --- | --- |
| 1 | F | 49 | 20 | LSCC; right middle and upper | Tumor;  tumor-adjacent tissue |
| 2 | M | 60 | Non-smoker | LSCC; right | Tumor;  tumor-adjacent tissue |
| 3 | F | 59 | 40 | LSCC; right | Tumor;  tumor-adjacent tissue |
| 4 | F | 46 | 30 | LSCC; right lower | Tumor;  tumor-adjacent tissue |
| 5 | F | 57 | 40 | LSCC; right middle and lower | Tumor;  tumor-adjacent tissue |
