# Supplementary material for: Ubiquitination-mediated molecular pathway alterations in human lung squamous cell carcinomas identified by quantitative ubiquitinomics
Source: Front Endocrinol (Lausanne). 2022 Sep 15;13:970843. doi: 10.3389/fendo.2022.970843 (PMC9520991; doi:10.3389/fendo.2022.970843)
Supplement: Supplementary file 1 [file DataSheet_1.zip › Supplemental Table 10_v5.doc]

Supplemental **Table 10. The function characteristics of 234 hub molecules**

| **Category** | **Term** | **PValue** | **Benjamini** |
| --- | --- | --- | --- |
| ***Cluster 1*** |  |  |  |
| KEGG_PATHWAY | Ribosome | 1.43E-05 | 7.23E-04 |
| GOTERM_BP_DIRECT | SRP-dependent cotranslational protein targeting to membrane | 3.43E-11 | 1.92E-08 |
| GOTERM_BP_DIRECT | viral transcription | 4.14E-09 | 6.95E-07 |
| GOTERM_BP_DIRECT | nuclear-transcribed mRNA catabolic process, nonsense-mediated decay | 8.62E-10 | 1.81E-07 |
| GOTERM_BP_DIRECT | translational initiation | 5.56E-09 | 8.48E-07 |
| GOTERM_BP_DIRECT | translation | 2.09E-06 | 1.17E-04 |
| GOTERM_CC_DIRECT | ribosome | 2.02E-07 | 4.00E-06 |
| GOTERM_BP_DIRECT | rRNA processing | 7.82E-06 | 3.75E-04 |
| GOTERM_MF_DIRECT | structural constituent of ribosome | 2.35E-06 | 1.14E-04 |
| ***Cluster 2*** |  |  |  |
| GOTERM_BP_DIRECT | cell-cell adhesion | 6.00E-18 | 1.01E-14 |
| GOTERM_CC_DIRECT | cell-cell adherens junction | 1.22E-21 | 9.18E-20 |
| GOTERM_MF_DIRECT | cadherin binding involved in cell-cell adhesion | 3.46E-23 | 5.62E-21 |
| ***Cluster 3*** |  |  |  |
| GOTERM_CC_DIRECT | cytosolic proteasome complex | 6.73E-03 | 3.88E-02 |
| GOTERM_CC_DIRECT | nuclear proteasome complex | 4.26E-03 | 2.68E-02 |
| GOTERM_MF_DIRECT | TBP-class protein binding | 2.80E-03 | 3.53E-02 |
| GOTERM_MF_DIRECT | proteasome-activating ATPase activity | 2.70E-03 | 3.59E-02 |
| ***Cluster 4*** |  |  |  |
| KEGG_PATHWAY | Biosynthesis of amino acids | 2.97E-03 | 3.11E-02 |
| KEGG_PATHWAY | Glycolysis / Gluconeogenesis | 1.96E-03 | 2.79E-02 |
| GOTERM_BP_DIRECT | luconeogenesis | 3.18E-04 | 1.06E-02 |
| GOTERM_BP_DIRECT | glycolytic process | 9.10E-05 | 3.72E-03 |
| GOTERM_BP_DIRECT | canonical glycolysis | 4.22E-08 | 4.72E-06 |
| ***Cluster 5*** |  |  |  |
| GOTERM_BP_DIRECT | positive regulation of ubiquitin-protein ligase activity involved in regulation of mitotic cell cycle transition | 7.95E-08 | 8.33E-06 |
| GOTERM_BP_DIRECT | negative regulation of ubiquitin-protein ligase activity involved in mitotic cell cycle | 4.08E-08 | 4.89E-06 |
| GOTERM_BP_DIRECT | NIK/NF-kappaB signaling | 1.98E-08 | 2.77E-06 |
| GOTERM_BP_DIRECT | Wnt signaling pathway, planar cell polarity pathway | 3.46E-10 | 9.68E-08 |
| GOTERM_BP_DIRECT | antigen processing and presentation of exogenous peptide antigen via MHC class I, TAP-dependent | 1.74E-07 | 1.33E-05 |
| GOTERM_BP_DIRECT | tumor necrosis factor-mediated signaling pathway | 6.35E-07 | 4.10E-05 |
| GOTERM_BP_DIRECT | regulation of cellular amino acid metabolic process | 2.58E-08 | 3.33E-06 |
| GOTERM_BP_DIRECT | positive regulation of canonical Wnt signaling pathway | 7.52E-07 | 4.67E-05 |
| GOTERM_BP_DIRECT | T cell receptor signaling pathway | 1.21E-07 | 1.01E-05 |
| GOTERM_BP_DIRECT | anaphase-promoting complex-dependent catabolic process | 1.16E-07 | 1.08E-05 |
| GOTERM_BP_DIRECT | negative regulation of canonical Wnt signaling pathway | 2.46E-06 | 1.29E-04 |
| KEGG_PATHWAY | Proteasome | 1.45E-04 | 4.17E-03 |
