# Supplementary material for: Ubiquitination-mediated molecular pathway alterations in human lung squamous cell carcinomas identified by quantitative ubiquitinomics
Source: Front Endocrinol (Lausanne). 2022 Sep 15;13:970843. doi: 10.3389/fendo.2022.970843 (PMC9520991; doi:10.3389/fendo.2022.970843)
Supplement: Supplementary file 1 [file DataSheet_1.zip › Supplemental Table 3_v5.doc]

**Supplemental Table 3. Functional characteristics of UPs identified with GO enrichment analysis of 627 UPs in LSCC.**

| **Category** | **Term** | **PValue** | **Benjamini** |
| --- | --- | --- | --- |
| ***Annotation Cluster 1*** |  |  |  |
| GOTERM_BP_DIRECT | regulation of cellular amino acid metabolic process | 4.14E-09 | 1.86E-06 |
| GOTERM_BP_DIRECT | Wnt signaling pathway, planar cell polarity pathway | 5.78E-11 | 3.90E-08 |
| GOTERM_BP_DIRECT | NIK/NF-kappaB signaling | 1.37E-08 | 4.12E-06 |
| GOTERM_BP_DIRECT | stimulatory C-type lectin receptor signaling pathway | 4.43E-09 | 1.71E-06 |
| GOTERM_BP_DIRECT | negative regulation of ubiquitin-protein ligase activity involved in mitotic cell cycle | 3.70E-08 | 7.67E-06 |
| GOTERM_BP_DIRECT | tumor necrosis factor-mediated signaling pathway | 2.96E-08 | 7.27E-06 |
| GOTERM_BP_DIRECT | anaphase-promoting complex-dependent catabolic process | 1.52E-07 | 2.27E-05 |
| GOTERM_BP_DIRECT | positive regulation of ubiquitin-protein ligase activity involved in regulation of mitotic cell cycle transition | 9.13E-08 | 1.45E-05 |
| GOTERM_BP_DIRECT | negative regulation of canonical Wnt signaling pathway | 1.57E-05 | 1.28E-03 |
| GOTERM_BP_DIRECT | positive regulation of canonical Wnt signaling pathway | 1.13E-06 | 1.22E-04 |
| GOTERM_BP_DIRECT | T cell receptor signaling pathway | 9.74E-07 | 1.09E-04 |
| ***Annotation Cluster 2*** |  |  |  |
| GOTERM_CC_DIRECT | cell-cell adherens junction | 3.72E-29 | 4.09E-27 |
| GOTERM_BP_DIRECT | cell-cell adhesion | 1.13E-23 | 3.05E-20 |
| GOTERM_MF_DIRECT | cadherin binding involved in cell-cell adhesion | 1.43E-29 | 5.95E-27 |
| ***Annotation Cluster 3*** |  |  |  |
| GOTERM_BP_DIRECT | nuclear-transcribed mRNA catabolic process, nonsense-mediated decay | 1.92E-07 | 2.72E-05 |
| GOTERM_BP_DIRECT | SRP-dependent cotranslational protein targeting to membrane | 3.56E-08 | 8.01E-06 |
| GOTERM_BP_DIRECT | translational initiation | 6.60E-06 | 6.14E-04 |
| GOTERM_BP_DIRECT | viral transcription | 7.74E-08 | 1.49E-05 |
| GOTERM_MF_DIRECT | structural constituent of ribosome | 1.68E-03 | 3.90E-02 |
| GOTERM_CC_DIRECT | ribosome | 3.32E-05 | 4.35E-04 |
| ***Annotation Cluster 4*** |  |  |  |
| GOTERM_BP_DIRECT | nucleotide-excision repair, DNA damage recognition | 6.40E-04 | 3.33E-02 |
| ***Annotation Cluster 5*** |  |  |  |
| GOTERM_CC_DIRECT | haptoglobin-hemoglobin complex | 9.95E-05 | 1.17E-03 |
| ***Annotation Cluster 6*** |  |  |  |
| GOTERM_BP_DIRECT | positive regulation of proteasomal protein catabolic process | 9.97E-05 | 6.87E-03 |
| GOTERM_CC_DIRECT | proteasome regulatory particle, base subcomplex | 1.29E-08 | 3.39E-07 |
| GOTERM_BP_DIRECT | positive regulation of RNA polymerase II transcriptional preinitiation complex assembly | 3.45E-06 | 3.45E-04 |
| GOTERM_MF_DIRECT | TBP-class protein binding | 3.27E-05 | 1.36E-03 |
| GOTERM_CC_DIRECT | nuclear proteasome complex | 1.70E-08 | 4.06E-07 |
| GOTERM_MF_DIRECT | proteasome-activating ATPase activity | 1.70E-07 | 1.76E-05 |
| GOTERM_CC_DIRECT | cytosolic proteasome complex | 1.21E-07 | 2.38E-06 |
| ***Annotation Cluster 7*** |  |  |  |
| GOTERM_CC_DIRECT | hemoglobin complex | 4.59E-03 | 3.37E-02 |
