# Supplementary material for: Ubiquitination-mediated molecular pathway alterations in human lung squamous cell carcinomas identified by quantitative ubiquitinomics
Source: Front Endocrinol (Lausanne). 2022 Sep 15;13:970843. doi: 10.3389/fendo.2022.970843 (PMC9520991; doi:10.3389/fendo.2022.970843)
Supplement: Supplementary file 1 [file DataSheet_1.zip › Supplemental Table 9_v5.doc]

**Supplemental Table 9. Hub molecules identified with six topological algorithms based on 627 Ups**

| **Topological algorithms** | **Number** | **Hub molecules** |
| --- | --- | --- |
| **Closeness** | 347 | GAPDH, HSP90AA1, RPS27A, UBA52, HSPA8, EEF1A1, VCP, HSP90AB1, GNB2L1, RHOA, PPP2CA, KRAS, RPS3, PKM, ENO1, TPI1, RPLP0, CCT8, CAV1, RPS2, RPS20, UBE2N, VCL, RPS16, YWHAE, POLR2B,HNRNPA2B1, TCP1, PSMA6, RPL11, CCT6A, PSMD4, RPL3, RAC1, RPL9, ANXA2, PSMC4, CD44, HSPB1, YWHAB, ANXA5, YWHAZ, PGK1, PSMD11, HNRNPK, PSMC5, ITGB1, RAD23B, ATP5B, RPL7A, ATP5A1, PSMC1, RPL12, PCBP1, UBA1, LMNA, ANXA1, IGF1R, RPS15, PRKDC, MYH9, PLEC, EIF2S3, H2AFX, ACTC1, RAD23A, YWHAQ, ILF2, VDAC1, YWHAG, TUBB4B, H2AFZ, ALDOA, NME2, TUBA1B, IQGAP1, TUBA4A, PRDX1, XRCC6, MYH10, HIST2H2AC, RPL18A, GJA1, ICAM1, PHGDH, USP7, LDHB, XRCC5, VAMP8, TARDBP, IKBKG, PFN1, GSN, SHC1, HIST1H4A, PPP1CB, HMGB1, RAC2, ACLY, VIM, NACA, PSMD3, PSMD8, SLC2A1, MAP2K2, H1F0, ADRM1, MSN, IMPDH2, PCNA, PLD1, MYL6, CALM2, SYK, SET, PCBP2, ITCH, ABCC1, TNPO1, LMNB1, YWHAH, JAK1, FTL, MYH11, DYNLL1, VDAC3, DDX58, FTH1, ADAM17, TLN1, SLC2A3, DSTN, H3F3B, CUL5, UBXN7, EPHA2, UBE4B, GNAI2, CUL4A, NOTCH3, CALD1, SFN, PGD, CDA, DNAJB1, UCHL1, UBA6, CAPN1, PSMD10, CSF1R, PRPF8, PGAM1, TPM1, NQO1, HNRNPM, APAF1, CLTB, RAB2A, ILF3, PIP5K1A, EPCAM, HCK, PFKFB3, PML, SLC3A2, UNC45A, OLR1, MIB1, PI4K2A, ACTN1, ATP6V1A, FASN, PIK3C2A, EPS15L1, AIMP1, HIST1H2BJ, S100A4, RDX, GNB1, HP, OGT, AHR, SNAP23, CLDN5, CTNND1, FCHO2, RBMX, GAPVD1, APOA1, EPN1, TPM2, KRT18, ST13, HIST2H3D, HBB, HUWE1, GNG12, FSCN1, GNAS, HIST1H1C, CAD, SLC16A3, S100A6, TPM4, HIP1, RBCK1, FGR, GARS, HNRNPF, LRP1, GNB2, TXNIP, SGTA, S100A9, EIF2AK2, UBXN1, FGB, BAG1, HIST1H2AC, USP25, HNRNPH2, H2AFJ, SLC1A5, S100A8, HBA2, COPS2, CA2, H2AFY, AHNAK, SDCBP, CASK, UBE2O, SPTA1, EWSR1, GNG5, MYO1E, DSP, GCLM, MYO1C, FES, RAB27A, HIST1H1E, HIST1H1B, SRPRB, TUBGCP3, ALCAM, BRAP, LRSAM1, NGLY1, LAMTOR1, CHP1, DSG2, YOD1, AQP4, NUP93, MAP2K3, RALY, HIST2H2AB, PTMA, GNAI3, SAP18, PFN2, EFNB1, HIST1H2BC, CBX3, IDE, ATP11A, DDIT4, CLIC4, LAP3, VAT1, FAM213A, ZPR1, PRKAR1A, CBX1, TRIP13, DDI2, NCF4, EIF4G2, SLC16A4, ARIH1, GOLGA7, FLOT2, H2AFY2, EHD2, TYMP, MST4, BCAP31, NUP155, TSNAX, PTRF, ATP1A1, MACF1, STX4, DCUN1D1, S100A11, PDCD10, ANO6, MYOF, ANP32E, CADM1, F13A1, UTRN, JOSD1, ARHGAP15, LAPTM5, ANKRD13A, IKZF3, INTS7, RTCB, PI4KA, COL6A1, ARHGAP9, AKR1B10, MVB12B, RAB25, TMEM30A, ACTBL2, S100A10, SYNPO, MYO1G, PNP, S100A2, TMPO, AUP1, PLSCR1, ANK1, SELENBP1, UBTF, PRKCDBP, ZDHHC5, GBP2, MT2A, MYO1B, SPG20, CLIC2, SFTPC, SLC16A1, PPAP2B, SLC7A5, PCID2, ATP2B4, HBD, SLC43A2, WRNIP1, NEDD8-MDP1, RPS10-NUDT3 |
| **Degree** | 268 | GAPDH, HSP90AA1, RPS27A, UBA52, HSPA8, EEF1A1, GNB2L1, VCP, HSP90AB1, RHOA, RPS3, CCT8, RPLP0, PPP2CA, RPS2, ENO1, RPS20, PKM, RPS16, TPI1, RAD23B, KRAS, POLR2B, TCP1, CCT6A, HNRNPA2B1, RPL11, VCL, RPL9, RPL3, RPL12, PSMA6, PSMC4, RAC1, CAV1, PSMC5, PSMD4, PSMD11, UBE2N, PSMC1, RAD23A, EIF2S3, HNRNPK, PGK1, YWHAE, RPL7A, ANXA2, YWHAZ, PCBP1, ILF2, CD44, RPL18A, ATP5A1, RPS15, YWHAB, ATP5B, HSPB1, PLEC, ACTC1, H2AFZ, ITGB1, ANXA1, UBA1, ADRM1, MYH9, TNPO1, LMNA, ANXA5, HIST2H2AC, H2AFX, MYH10, PSMD8, ALDOA, ITCH, NACA, XRCC6, VAMP8, IGF1R, IQGAP1, PCBP2, PRKDC, YWHAG, YWHAQ, LDHB, ICAM1, PRDX1, TUBB4B, HNRNPM, HIST1H4A, NME2, TUBA1B, TLN1, XRCC5, HIST1H2BJ, ILF3, TUBA4A, SHC1, TARDBP, GSN, H1F0, VIM, USP7, PCNA, UBXN7, ACLY, RAC2, VDAC1, AIMP1, IMPDH2, GJA1, SLC2A1, HIST1H1C, ACTN1, TPM1, PHGDH, PFN1, DYNLL1, DSTN, MYL6, CUL4A, UBA6, PPP1CB, PSMD3, UBE4B, TPM2, MAP2K2, HIST2H3D, RBMX, HIST1H2AC, H3F3B, H2AFY, MSN, LMNB1, GNAI2, SLC2A3, YWHAH, HUWE1, H2AFJ, HP, SYK, PRPF8, HIST1H1E, MYH11, PLD1, TPM4, HIST1H1B, SNAP23, HNRNPF, CTNND1, CALM2, HMGB1, GNB1, COPS2, SFN, CUL5, JAK1, ABCC1, HIST2H2AB, HIST1H2BC, CBX3, FTL, FTH1, PIP5K1A, HCK, CALD1, ATP6V1A, PGD, CBX1, MYO1E, IKBKG, RBCK1, RDX, SRPRB, FGR, NQO1, GARS, CSF1R, APOA1, SAP18, PFKFB3, LAMTOR1, GNG12, CDA, PGAM1, AHNAK, HNRNPH2, CLTB, EPCAM, FGB, SET, CAD, PSMD10, GNB2, PML, SLC3A2, EPHA2, UNC45A, VDAC3, DDX58, UBE2O, EWSR1, PIK3C2A, ANO6, RAB2A, GNAI3, APAF1, HBB, HBA2, GNAS, UBXN1, EIF2AK2, CAPN1, MYO1C, H2AFY2, RAB27A, DNAJB1, FASN, EPS15L1, OLR1, FCHO2, ATP11A, RALY, GNG5, KRT18 ,EFNB1, DSP, S100A4, EPN1, SPTA1, TMEM30A, SDCBP, GAPVD1, ARIH1, MIB1, SLC1A5, S100A6, LRSAM1, SLC16A3, LRP1, CLDN5, BCAP31, ADAM17, BAG1, UCHL1, DSG2, NCF4, ST13, PRKAR1A, HIP1, PTRF, OGT, USP25, DDIT4 ,NOTCH3, STX4, PTMA, RAB25, S100A9, S100A8, YOD1, NGLY1, SGTA, CLIC4, TUBGCP3, PFN2, PI4K2A, NEDD8-MDP1, RPS10-NUDT3 |
| **EPC** | 278 | GAPDH, HSP90AA1, HSPA8, RPS27A, UBA52, GNB2L1, EEF1A1, RPS3, RPLP0, RPS2, HSP90AB1, RPS16, RPS20, PSMA6, CCT8, VCP, PPP2CA, RPL3, RPL9, RPL11, PSMC4, HNRNPA2B1, RPL12, PSMC5, PSMC1, PSMD4, TCP1, EIF2S3, RPL7A, PKM, POLR2B, PSMD11, ENO1, CCT6A, RAD23B, RPL18A, TPI1, PGK1, RAD23A, HNRNPK, RPS15, UBE2N, ATP5B, NACA, ADRM1, ATP5A1, PSMD8, PLEC, TNPO1, PCBP1, RHOA, ILF2, YWHAB, YWHAZ, YWHAE, KRAS, HSPB1, ANXA2, IMPDH2, UBA1, RAC1, PCBP2, NME2, HNRNPM, TUBA1B, TUBB4B, AIMP1, ILF3, PSMD3, VCL, ANXA5, CAV1, H2AFZ, TUBA4A, PRKDC, XRCC6, IGF1R, YWHAQ, H2AFX, ITCH, TARDBP, XRCC5, PRDX1, LDHB, ACTC1, HIST2H2AC, YWHAG, ALDOA, MYH9, LMNA, ACLY, HIST1H4A, UBXN7, SRPRB, UBA6, VDAC1, PSMD10, ITGB1, PCNA, ANXA1, IQGAP1, PHGDH, CAD, PFN1, CD44, USP7, MAP2K2, PPP1CB, HIST1H2BJ, COPS2, H1F0, PRPF8, VIM, RBMX, MYH10, SET, GARS, CUL4A, GSN, YWHAH, SFN, IKBKG, DSTN, TLN1, SLC2A1, H2AFJ, GJA1, HIST1H1C, HNRNPF, PGAM1, HMGB1, HNRNPH2, HIST2H3D, H3F3B, SHC1, CUL5, UBE4B, HUWE1, MYL6, H2AFY, MSN, HIST1H1E, HIST1H2AC, APAF1, CALM2, PIP5K1A, HIST1H1B, RBCK1, GNAI2, PGD, LMNB1, HIST2H2AB, JAK1, ACTN1, DYNLL1, RALY, PFKFB3, MYH11, GNB1, RAC2, ICAM1, SLC2A3, SAP18, TPM1, CAPN1, UBXN1, FTL, HIST1H2BC, DNAJB1, VDAC3, UCHL1, SYK, GNB2, HBA2, VAMP8, TPM2, ATP6V1A, MIB1, BAG1, CBX3, EIF2AK2, EWSR1, FTH1, EPHA2, ABCC1, CBX1, ADAM17, PML, TPM4, UNC45A, CDA, CALD1, PLD1, LRSAM1, ST13, CLTB, GNG12, FASN, H2AFY2, NGLY1, EPS15L1, EIF4G2, SGTA, SLC16A3, DDX58, ARIH1, FGB, PIK3C2A, GNAS, AHR, UBE2O, HCK, CTNND1, GNG5, NOTCH3, USP25, RDX, GAPVD1, GNAI3, DDI2, FCHO2, FSCN1, RTCB, S100A4, SLC3A2, MYO1E, NQO1, KRT18, EPCAM, EPN1, CSF1R, HP, OGT, YOD1, HIP1, TUBGCP3, PFN2, MYO1C, DDIT4, OLR1, SNAP23, BRAP, FGR, MAP2K3, AHNAK, PI4K2A, APOA1, RAB2A, S100A6, TRIP13, HBB, SDCBP, PTMA, TXNIP, IDE, NCF4, DCUN1D1, DSP, PRKAR1A, LAMTOR1, FES, LRP1, NUP93, UBTF, VAT1, SLC16A4, CLDN5, ACTBL2, DSG2, S100A9, GCLM, SLC1A5, EFNB1, ANP32E, S100A11, ATP11A, NEDD8-MDP1, RPS10-NUDT3 |
| **MCC** | 263 | RPLP0, GNB2L1, RPS2, RPS3, RPS16, RPL3, RPL11, EEF1A1, RPL9, RPL7A, RPL12, RPL18A, PSMC5, PSMA6, CCT8, PSMD11, PSMC1, CCT6A, TCP1, RPS20, EIF2S3, PSMC4, PPP2CA, RPS27A, HSPA8, UBA52, TNPO1, NACA, RPS15, PLEC, HSP90AB1, RAD23B, PSMD4, HSP90AA1, HNRNPA2B1, RAD23A, ADRM1, GAPDH, AIMP1, POLR2B, PSMD8, HIST1H1C, HIST1H4A, H3F3B, HIST2H3D, HIST1H2BJ, HIST1H2BC, HIST1H1E, HIST1H1B, HIST2H2AB, HIST2H2AC, HIST1H2AC, H1F0, H2AFY, SRPRB, H2AFZ, IMPDH2, ATP5A1, PGK1, ENO1, ATP5B, PSMD3, H2AFJ, PKM, VCP, TPI1, COPS2, H2AFY2, H2AFX, NME2, PSMD10, PCBP1, CBX3, PCBP2, HNRNPK, UBA1, ITCH, CBX1, PRDX1, LDHB, ALDOA, PGAM1, HNRNPM, ILF2, UBXN7, PGD, RBMX, ILF3, HNRNPF, UBE2N, UBA6, ACTN1, MYH9, MYH11, TPM1, VCL, MYL6, CALD1, HNRNPH2, TUBA1B, MYH10, ACTC1, TPM2, TPM4, PRPF8, HUWE1, YWHAE, RBCK1, CUL5, UBE2O, LRSAM1, YWHAB, TUBB4B, YWHAZ, YWHAG, TARDBP, YWHAQ, PFKFB3, KRAS, MAP2K2, SFN, RHOA, GARS, SLC2A1, TLN1, YWHAH, CAD, ACLY, VAMP8, ANXA2, PPP1CB, USP7, SLC2A3, RALY, CLTB, EPS15L1, HIP1, EPN1, HSPB1, LMNB1, TUBA4A, PHGDH, VDAC1, CUL4A, EWSR1, PIK3C2A, SLC16A3, XRCC6, XRCC5, DSTN, PLD1, SNAP23, ANO6, OLR1, ATP11A, PCNA, LAMTOR1, TMEM30A, FCHO2, GAPVD1, RAC1, ANXA5, CD44, CAV1, IQGAP1, IGF1R, GNB1, GNB2, GNG12, GNG5, PRKDC, GNAI2, GNAI3, DYNLL1,UBE4B, LMNA, GSN, ANXA1, FTL, SAP18, ITGB1, EPHA2, FGB, GNAS, DDI2, SDCBP, DDIT4, TUBGCP3, VAT1, RAC2, PFN1, ICAM1, SET, VIM, UBXN1, JAK1, MSN, SYK, NGLY1, HBA2, SHC1, DNAJB1, APAF1, CAPN1, PIP5K1A, GJA1, IKBKG, FTH1, USP25, PFN2, EPCAM, UBTF, CDA, S100A4, HP, HBB, CSF1R, EIF2AK2, MYO1E, GOLGA7, RDX, YOD1, CALM2, CTNND1, ACTBL2, HMGB1, UCHL1, DCUN1D1, ADAM17, AHNAK, S100A10, SLC16A4, STX4, S100A11, DDX58, STX3, STXBP2, HCK, SLC3A2, NCF4, ST13, ATP6V1A, S100A6, ABCC1, NQO1, MIB1, RAB27A, ARHGAP9, FGR, VDAC3, GCLM, BAG1, SLC1A5, CPNE1, CPNE3, VAMP5, ALCAM, ARL8A, UNC45A, NOTCH3, EFNB1, RPS10-NUDT3, NEDD8-MDP1 |
| **MNC** | 232 | HSP90AA1, RPS27A, UBA52, HSPA8, EEF1A1, GNB2L1, VCP, HSP90AB1, RHOA, RPS3, CCT8, RPLP0, PPP2CA, RPS2, RPS20, ENO1, PKM, RPS16, TPI1, RAD23B, KRAS, TCP1, CCT6A, POLR2B, RPL11, HNRNPA2B1, RPL9, RPL3, VCL, RPL12, PSMA6, PSMC4, PSMD4, PSMD11, RAC1, CAV1, PSMC5, UBE2N, PSMC1, PGK1, EIF2S3, RAD23A, RPL7A, HNRNPK, YWHAZ, ANXA2, PCBP1, RPL18A, ATP5A1, ILF2, YWHAE, YWHAB, ATP5B, CD44, RPS15, HSPB1, ACTC1, H2AFZ, ITGB1, PLEC, ANXA1, ADRM1, MYH9, LMNA, ANXA5, HIST2H2AC, UBA1, H2AFX, PSMD8, ALDOA, MYH10, NACA, XRCC6, YWHAQ, IQGAP1, TNPO1, PCBP2, YWHAG, LDHB, TUBB4B, PRKDC, HNRNPM, HIST1H4A, ITCH,PRDX1, TUBA1B, HIST1H2BJ, ILF3, TUBA4A, SHC1, TARDBP, ICAM1, GSN, H1F0, TLN1, XRCC5, PCNA, RAC2, VDAC1, IGF1R, NME2, IMPDH2, HIST1H1C, ACTN1, USP7, UBXN7, ACLY, PFN1, GJA1, SLC2A1, VIM, TPM1, UBA6, PPP1CB, AIMP1, PSMD3, TPM2, MAP2K2, MYL6, HIST1H2AC, H3F3B, HIST2H3D, PHGDH, MSN, LMNB1, DYNLL1, RBMX, GNAI2, SLC2A3, YWHAH, H2AFJ, PRPF8, H2AFY, HIST1H1E, MYH11, DSTN, HIST1H1B, HP, CUL4A, UBE4B, SYK, PLD1, HMGB1, GNB1, HNRNPF, VAMP8, COPS2, TPM4, CALM2, SNAP23, HIST2H2AB, HUWE1, HIST1H2BC, FTL, FTH1, HCK, JAK1, PGD, SFN, CUL5, CALD1, NQO1, PFKFB3, IKBKG, RBCK1, PGAM1, SRPRB, FGR, SET, CAD, PSMD10, GNB2, EPHA2, CTNND1, HNRNPH2, CBX3, CLTB, DDX58, GARS, FGB, PIK3C2A, CBX1, APOA1, LAMTOR1, GNG12, CDA, HBB, SLC3A2, RDX, VDAC3, EIF2AK2, CAPN1, GNAI3, ABCC1, H2AFY2, APAF1, DNAJB1, HBA2, GNAS, AHNAK, UBXN1, ATP6V1A, EPCAM, CSF1R, GNG5 ,FASN, EPN1, EPS15L1, OLR1, UNC45A, PIP5K1A, RALY, UBE2O, SLC1A5, LRSAM1, MYO1C, SLC16A3, MYO1E, ADAM17, BAG1, KRT18, UCHL1, DSG2, S100A4, NCF4, FCHO2 ,HIP1, ATP11A, TMEM30A, S100A6, RPS10-NUDT3, NEDD8-MDP1 |
| **Raliality** | 334 | GAPDH, HSP90AA1, RPS27A, HSPA8, UBA52, VCP, EEF1A1, RHOA, HSP90AB1, KRAS, GNB2L1, PPP2CA, TPI1, PKM, ENO1, CAV1, RPS3, YWHAE, RPLP0, UBE2N, RPS2, VCL, ANXA5, CCT8, ANXA2, HSPB1, YWHAB, CD44, RPS16, RPS20, PSMA6, TCP1, HNRNPA2B1, POLR2B, PSMD4, RAC1, YWHAZ, ITGB1, RPL11, RPL3, CCT6A, RPL9, IGF1R, PSMC4, PGK1, ATP5B, LMNA, PRKDC, ATP5A1, UBA1, VDAC1, HNRNPK, YWHAQ, PSMD11, ANXA1, RPL7A, MYH9, PCBP1, TUBB4B, YWHAG, H2AFX, RPS15, PSMC5, TUBA1B, ACTC1, NME2, PSMC1, TUBA4A, PLEC, ALDOA, IKBKG, IQGAP1, PRDX1, RPL12, XRCC6, H2AFZ, RAD23B, ILF2, PHGDH, GJA1, USP7, HMGB1, EIF2S3, MYH10, ICAM1, PFN1, PPP1CB, SHC1, RAD23A, HIST2H2AC, RAC2, PSMD3, XRCC5, LDHB, TARDBP, GSN, HIST1H4A, VIM, ACLY, MAP2K2, MSN, SLC2A1, SET, VAMP8, CALM2, PLD1, SYK, ADAM17, MYL6, H1F0, IMPDH2, ABCC1, RPL18A, NACA, PCNA, VDAC3, FTL, PSMD8, DDX58, JAK1, YWHAH, MYH11, NOTCH3 ,FTH1, LMNB1, EPHA2, ADRM1, UCHL1, SLC2A3, DNAJB1, DYNLL1, CUL5, H3F3B, CDA, TLN1, CAPN1, PCBP2, PGD, GNAI2, CALD1, DSTN, RAB2A, PI4K2A, CSF1R, SFN, APAF1, ITCH, PGAM1, PSMD10, UBE4B, OLR1, TNPO1, NQO1, MIB1, PML, UBXN7, SLC3A2, PFKFB3, CUL4A, CLTB, FASN, UNC45A, EPCAM, S100A4, AHR, UBA6, HCK, OGT, CLDN5, EPS15L1, PRPF8, PIP5K1A, ATP6V1A, PIK3C2A, TPM1, RDX, FSCN1, KRT18, ST13, GAPVD1, SLC16A3, ACTN1, EPN1, FCHO2, HBB, GNB1, SNAP23, APOA1, GNAS, CTNND1, TXNIP, HP, HNRNPM, ILF3, S100A6, LRP1, GNG12, HIP1, S100A9, CA2, SGTA, CAD, FGR, AIMP1, RBMX, TPM2, S100A8, HIST1H2BJ, GNB2, BAG1, SLC1A5, HUWE1, USP25, RBCK1, HIST2H3D, TPM4, EIF2AK2, GARS, CASK, UBXN1, SDCBP, FGB, CHP1, HNRNPF, GCLM, ALCAM, BRAP, HIST1H1C, HBA2, SPTA1, AHNAK, HNRNPH2, FES, GNG5, TUBGCP3, MYO1C, AQP4, EWSR1, RAB27A, DSP, NGLY1, MYO1E, YOD1, DSG2, UBE2O, H2AFJ, NUP93, HIST1H2AC, COPS2, LRSAM1, MAP2K3,H2AFY, FAM213A, LAMTOR1, SRPRB, PFN2, IDE, EFNB1, VAT1, HIST1H1E, HIST1H1B, PTMA, GNAI3, LAP3, RALY, DDIT4, ZPR1, CLIC4, PRKAR1A, ATP11A, SLC16A4, FLOT2, SAP18, TRIP13, TYMP, HIST2H2AB, MST4, NCF4, GOLGA7, EHD2, EIF4G2, CBX3, MACF1, HIST1H2BC, BCAP31, TSNAX, DDI2, NUP155, ATP1A1, CBX1, PDCD10, STX4, ARIH1, H2AFY2, PTRF, MYOF, F13A1, S100A11, DCUN1D1, ANKRD13A, UTRN, CADM1, ANP32E, JOSD1, IKZF3, ANO6, ARHGAP15, PI4KA, LAPTM5, INTS7, COL6A1, RTCB, SYNPO, ARHGAP9, AKR1B10, MVB12B, PNP, AUP1, S100A10, RAB25, ACTBL2, MYO1G, TMEM30A, ANK1, PLSCR1, SELENBP1, ZDHHC5, TMPO, S100A2, GBP2, MT2A, PRKCDBP, RPS10-NUDT3, NEDD8-MDP1 |
| **Overlap** | 234 | PFN2, CD44, ANXA2, HCK, ENO1, VCL, RPS2, HSPB1, GAPVD1, VAMP8, NCF4, EIF2AK2, RAC1, FTH1, YWHAH, TUBA4A, ANXA1, EWSR1, RDX, EIF2S3, TLN1, HIST1H1B, H2AFJ, RBMX, PSMC5, CBX3, RPLP0, PGAM1, IKBKG, PCBP1, GSN, HIST2H3D, ATP5A1, YWHAG, GNAI3, FGR, TCP1, RBCK1, LMNB1, ITGB1, COPS2, MSN, DNAJB1, MAP2K2, PSMC4, PKM, RPL7A, VIM, ADAM17, JAK1, PSMD8, CTNND1, UBA1, GJA1, GNAI2, TPM2, ITCH, PIP5K1A, SHC1, SET, PCNA, TPM4, RAD23A, ILF2, CUL4A, DDX58, EFNB1, NME2, S100A4, RPS20, EPHA2, IMPDH2, HNRNPA2B1, MYH10, CUL5, PHGDH, RAC2, APAF1, RPL11, MYH11, TUBB4B, YOD1, USP25, RALY, CBX1, XRCC5, UCHL1, OLR1, GNAS, HNRNPF, ATP6V1A, PFN1, ABCC1, RAD23B, HNRNPK, NQO1, SNAP23, H2AFY2, PCBP2, TNPO1, TPM1, GNB1, HIST1H1E, PRKDC, TPI1, CALD1, ATP11A, UBA6, DSTN, YWHAE, ACLY, NACA, SYK, PFKFB3, POLR2B, RPL12, IQGAP1, PSMA6, YWHAQ, USP7, SFN, CAD, LMNA, VDAC1, VDAC3, SLC2A3, HIST2H2AB, HNRNPH2, GNG5, ACTN1, PRPF8, PSMD3, MYH9, CLTB, HNRNPM, GAPDH, ATP5B, XRCC6, RPL18A, DYNLL1, LAMTOR1, EPS15L1, UBE2O, HIP1, NGLY1, PSMD11, HIST1H1C, AIMP1, S100A6, IGF1R, UBA52, CCT8, GNB2L1, HIST1H4A, ICAM1, RPS27A, RPS3, RPL3, CAV1, LDHB, CSF1R, H2AFZ, HIST1H2BC, ILF3, CAPN1, H2AFX, CDA, GNB2, SLC16A3, ACTC1, PRDX1, H3F3B, FGB, ADRM1, PLEC, UNC45A, SRPRB, PPP1CB, PGD, EPN1, HSP90AB1, VCP, SLC3A2, RPS16, ALDOA, KRAS, HIST1H2AC, CALM2, GNG12, PSMD10, PLD1, BAG1, HIST1H2BJ, UBE4B, RHOA, FCHO2, CCT6A, HSPA8, PSMC1, UBXN7, SLC1A5, HIST2H2AC, RPL9, MYL6, TARDBP, ANXA5, LRSAM1, PSMD4, SLC2A1, UBXN1, EEF1A1, H1F0, GARS, EPCAM, AHNAK, HBB, TUBA1B, PPP2CA, HSP90AA1, HBA2, HMGB1, UBE2N, FTL, YWHAB, YWHAZ, PGK1, PIK3C2A, MYO1E, RPS15, HUWE1, H2AFY, HP, NEDD8-MDP1, RPS10-NUDT3 |
